# Identifying predictors of ventral hernia recurrence: systematic review and meta-analysis

**DOI:** 10.1093/bjsopen/zraa071

**Published:** 2021-04-11

**Authors:** S G Parker, S . Mallett, L Quinn, C P J Wood, R W Boulton, S Jamshaid, M . Erotocritou, S . Gowda, W . Collier, A A O Plumb, A C J Windsor, L Archer, S Halligan

**Affiliations:** Abdominal Wall Unit, Department of Surgery, University College Hospital, London, UK; Institute of Applied Health Research, University of Birmingham, Birmingham, UK; Institute of Applied Health Research, University of Birmingham, Birmingham, UK; University College London Medical School, London, UK; Abdominal Wall Unit, Department of Surgery, University College Hospital, London, UK; Abdominal Wall Unit, Department of Surgery, University College Hospital, London, UK; Abdominal Wall Unit, Department of Surgery, University College Hospital, London, UK; Abdominal Wall Unit, Department of Surgery, University College Hospital, London, UK; Abdominal Wall Unit, Department of Surgery, University College Hospital, London, UK; Abdominal Wall Unit, Department of Surgery, University College Hospital, London, UK; Centre of Medical Imaging, University College Hospital, London, UK; Abdominal Wall Unit, Department of Surgery, University College Hospital, London, UK; Centre for Prognosis Research, School of Primary, Community and Social Care, Keele University, Keele, UK; Centre of Medical Imaging, University College Hospital, London, UK

## Abstract

**Background:**

Ventra hernias are increasing in prevalence and many recur despite attempted repair. To date, much of the literature is underpowered and divergent. As a result there is limited high quality evidence to inform surgeons succinctly which perioperative variables influence postoperative recurrence. This systematic review aimed to identify predictors of ventral hernia recurrence.

**Methods:**

PubMed was searched for studies reporting prognostic data of ventral hernia recurrence between 1 January 1995 and 1 January 2018. Extracted data described hernia type (primary/incisional), definitions of recurrence, methods used to detect recurrence, duration of follow-up, and co-morbidity. Data were extracted for all potential predictors, estimates and thresholds described. Random-effects meta-analysis was used. Bias was assessed with a modified PROBAST (Prediction model Risk Of Bias ASsessment Tool).

**Results:**

Screening of 18 214 abstracts yielded 274 individual studies for inclusion. Hernia recurrence was defined in 66 studies (24.1 per cent), using 41 different unstandardized definitions. Three patient variables (female sex, age 65 years or less, and BMI greater than 25, 30, 35 or 40 kg/m^2^), five patient co-morbidities (smoking, diabetes, chronic obstructive pulmonary disease, ASA grade III–IV, steroid use), two hernia-related variables (incisional/primary, recurrent/primary), six intraoperative variables (biological mesh, bridged repair, open *versus* laparoscopic surgery, suture *versus* mesh repair, onlay/retrorectus, intraperitoneal/retrorectus), and six postoperative variables (any complication, surgical-site occurrence, wound infection, seroma, haematoma, wound dehiscence) were identified as significant prognostic factors for hernia recurrence.

**Conclusion:**

This study summarized the current evidence base for predicting ventral hernia recurrence. Results should inform best practice and future research.

## Teaser

To identify predictors of ventral hernia recurrence, prognostic data were extracted from 20 years of available literature and subsequently meta-analysed. New knowledge is presented regarding patient demographics, hernia characteristics, intraoperative factors and postoperative variables that predispose to recurrence. This review should inform best practice and future research.

## Introduction

Ventral hernia is defined as an abnormal protrusion of abdominal contents through a defect in the anterior abdominal wall. The prevalence of ventral hernia is increasing in the West^1^^,^^2^, due mainly to the obesity epidemic and an ageing population subjected to ever more abdominal surgery^1^. Larger, more complex ventral hernias are also increasingly prevalent, and present a significant surgical challenge^3^ requiring carefully planned elective repair. Indeed, recurrence rates after repair are reported as 15–40 per cent^4^^,^^5^, indicating that surgery can be ineffective, subjecting patients to the risks of major abdominal surgery for no long-term benefit.

Hernia recurrence is an extremely important postoperative outcome and assesses surgical efficacy. The ability to predict recurrence accurately would have considerable clinical utility, allowing surgeons to make better-informed decisions with their patients as to when, and when not, to operate. A systematic review was undertaken to provide guidance on when co-morbidity or hernia complexity might preclude repair.

To date, there is an abundance of literature assessing ventral hernia repair that describes the preoperative, intraoperative and postoperative variables that may be associated with recurrence. However, publications frequently have small cohorts, vary in study design, are single-centre, and report divergent results^6–8^. This frustrates the interpretation of current evidence, and findings are difficult to apply in clinical practice. Consequently, surgeons have limited guidance regarding when not to operate, and evidence for optimal repair is lacking. Consequently, a comprehensive prognostic systematic review of the published literature was performed to identify potential predictors of hernia recurrence after elective ventral hernia repair. By using subsequent meta-analysis to synthesize these data, the aim was to identify those predictors significantly associated with postoperative recurrence, from the whole range of published literature.

## Methods

This systematic review was reported according to the PRISMA statement^9^. Ethical permission is not required by the authors’ institution for systematic reviews of available primary literature. The protocol was registered with PROSPERO, the international prospective register of systematic reviews (CRD42016043071).

### Inclusion and exclusion criteria

#### Inclusion criteria for studies

The aim was to identify studies reporting hernia recurrence in patients following ventral hernia repair with curative intent between 1 January 1995 and 1 January 2018. Studies with fewer than 10 patients in any individual study group were excluded, as such data are likely to be subject to small study bias. Only English-language studies were included.

#### Target condition

The target condition was surgical ventral hernia repair with curative intent. All different ventral hernia morphologies were eligible, as were all Ventral Hernia Working Group (VHWG^10^) grades. Studies describing femoral and/or inguinal hernias (groin hernias) were excluded. Emergency hernia repair was, in general, excluded. However, as much of the primary literature includes a small proportion of patients undergoing emergency surgery, the study was eligible if this proportion was less than 10 per cent. Studies of primary closure after damage control laparotomy were excluded. However, patients having elective ventral hernia repair after primary closure from damage laparotomy were eligible, as were studies of those having elective repair with bridging repair (failure to establish primary fascial closure). Studies in which a proportion of patients had abdominal wall defects repaired with a bridging mesh after abdominal wall tumour excision were eligible. Parastomal hernias were excluded. Studies with concomitant bowel resection were included (as this is often intended) as long as the primary surgical intention was ventral hernia repair. Studies with concomitant gastrointestinal tumour removal or bariatric surgery were excluded.

#### Participants

Studies of adult participants were included. Paediatric studies (defined as 18 years or less) were excluded, as these are not representative of ‘typical’ patients with ventral hernia.

#### Follow-up

No minimum length of follow-up was stipulated.

#### Comparators

No restriction was placed on any study comparator group (such as operative technique, mesh type, position or mesh).

### Search strategy and string

The PubMed database was searched from 1 January 1995 to 1 January 2018. The search was limited using the terms ‘adult 19+’ and ‘human studies’, and to publications written in English. Two different search strings were combined to identify relevant articles for both ventral hernia repair and postoperative recurrence.

Search strategy 1 identified studies of ventral hernia disease including complex disease, and studies of surgical techniques used for hernia repair. To identify studies of ventral hernia disease, MeSH (Medical Subject Heading) terms ‘hernia’, ‘abdominal hernia’, ‘umbilical hernia’ and ‘ventral hernia’ were used, combined with keywords for ventral hernia repair. To identify studies of surgical techniques, MeSH terms: ‘general surgery’, ‘reconstructive surgical procedures’ and ‘surgical mesh’ were used. This was combined with keywords for specialized abdominal wall reconstructive techniques.

Search strategy 2 identified studies predicting ventral hernia recurrence. MeSH terms ‘ventral hernia’ and ‘abdominal wall hernia’ were combined with keywords for prognostic studies.

The complete search string is shown in *Appendix S1*.

### Citation management and screening

Identified citations were stored in an Excel^®^ spreadsheet (Microsoft Excel^®^ for Mac 2011 version 14.5.9; Microsoft, Redmond, WA, USA), which was subsequently uploaded into a reference manager able to access online original articles directly (Mendeley Desktop version 1.17 for Windows XP and Mac OS X; Mendeley, London, UK). After search filters had been applied and duplicates excluded, citations were divided chronologically into two groups that were screened separately for studies potentially containing data on ventral hernia recurrence: interventional studies, cohort studies, observational/database studies. Clearly unsuitable articles were discarded, and ‘uncertain’ or ‘definitely possible’ articles were retained. The two latter groups were combined and three researchers then screened titles and abstracts independently to identify ventral hernia studies with prognostic data. Any discrepancies were settled by face-to-face discussion between the three researchers, who then examined the full texts and categorized studies by their methodological design as follows: RCTs; prospective interventional/cohort studies; retrospective interventional/cohort studies; observational/database studies. Any article for which uncertainty persisted was resolved by face-to-face discussion with senior authors. An exclusion log was kept at all stages.

### Data extraction

Included studies were scrutinized for prognostic data of ventral hernia recurrence. Data were extracted for all potential predictors from each article; for each predictor, risk estimates (2×2 tables, odds ratios (ORs), hazard ratios (HRs), adjusted ORs, relative risk ratios (RRs)), and thresholds were recorded. Where overlapping articles included data for the same predictors from the same patients, data were included only from the study describing the larger cohort. Confidence intervals and *P* values were extracted for all estimates, where available. Extracted predictors were grouped into preoperative, intraoperative and postoperative subcategories. For each study, the definition and method(s) used to identify hernia recurrence, and the mean time to recurrence, were extracted. Data from interventional trials tended to be 2×2 tables, whereas data from larger database studies tended to be univariable and/or multivariable ORs.

### Study characteristics and risk of bias

In addition to the preoperative, intraoperative and postoperative predictors, data relating to study setting (multicentre *versus* single-centre), country of publication, publication date, recruitment dates, number of patients included, severity of ventral hernia disease, and whether the study included primary or incisional hernias, or both, were also extracted. Severe disease was classified as a hernia with a width exceeding 10 cm and/or a contaminated hernia, graded as VHWG grade 4. Studies were scored as containing patients with severe disease only, mild disease only, or both mild and severe disease. Whether studies included participants with multiple grades or were restricted to severe disease was also recorded.

Risk of bias was assessed for individual studies using an adapted version of the PROBAST (Prediction model Risk Of Bias ASsessment Tool)^11^. PROBAST was developed to determine bias in published prediction models. As few prognostic models have been published for ventral hernia disease, PROBAST was adapted for detection of bias from all study designs. This bias tool was categorized according to study participants, extracted predictors, the definitions and detection of hernia recurrence, and according to statistical analysis (*Appendix S2*).

All data were stored using Microsoft Excel^®^.

### Statistical analysis

Because it was anticipated that data would be heterogeneous, predictor association with recurrence was sought rather than precise estimates of strength or interpredictor comparison. It was anticipated that study designs would include different definitions of recurrence, follow-up and patient populations that would cause variation in predictor estimates. Accordingly, meta-analysis would reflect general evidence across all studies rather than providing precise estimates regarding specific definitions, situations, measurements and thresholds. Most results could be extracted as 2×2 tables or univariable ORs; 2×2 results were converted into ORs for meta-analysis. Only OR results were available sufficiently to allow meta-analysis using the ‘metan’ command in STATA^®^ 14.2 (StataCorp, College Station, TX, USA).

Each study was included only once in each meta-analysis for a particular variable, to ensure that patients were included only once. A study could be included in each subgroup within a predictor, for example where different thresholds of a predictor value were meta-analysed separately. To exclude predictors with data insufficient for meaningful meta-analysis, predictors available from fewer than five primary studies were excluded, except those predictors considered ‘clinically important’.. Meta-analysis was considered for all predictors described in five or more individual studies if results were not considered heterogeneous based on visual inspection. A random-effects meta-analysis used the methods of DerSimonian and Laird, with the estimate of heterogeneity taken from the inverse-variance fixed-effect model^12^. Forest plots were used to present meta-analysis summaries across predictors and to present individual study results for each predictor. These plots indicate data characteristics including event, method of hernia recurrence detection, and whether incisional hernia, primary hernia, or both incisional and primary hernia populations, were included.

## Results

**Fig. 1:**
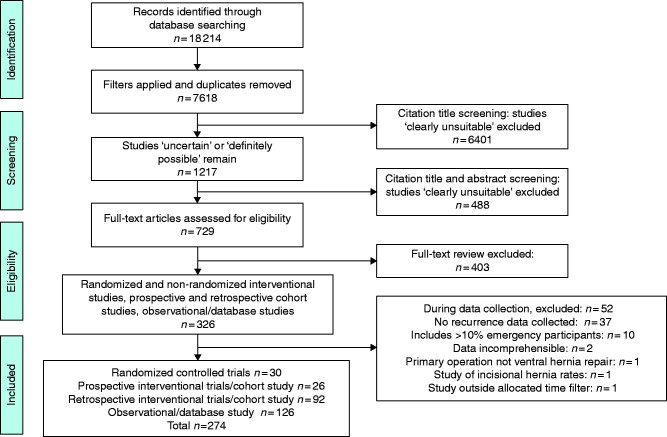
PRISMA diagram **showing selection of studies for review**

The PRISMA flow diagram is shown in *Fig. 1*. In total, 18 214 abstracts were identified, and 729 full texts were assessed for eligibility. Ultimately, prognostic data were extracted from 274 included manuscripts and 275 studies, with one manucript reporting two studies^13^; 31 RCTs (10.9 per cent), 26 prospective interventional or cohort studies (9.5 per cent), 92 retrospective interventional or cohort studies (33.6 per cent), and 126 database analyses (46.0 per cent). Most studies included originated from North America (137 of 274, 50.0 per cent), with 115 European (42.3 per cent). Of the 274 studies, 212 (77.4 per cent) were single-centre, 63 (23.0 per cent) were multicentre, and one study^13^ (0.4 per cent) presented both multicentre and single-centre data. Preoperative, intraoperative and postoperative prognostic factors were reported in 136 (35.2 per cent), 204 (52.8 per cent) and 46 (11.9 per cent) articles respectively. Regarding hernia type; 129 studies (47.1 per cent) assessed primary and incisional ventral hernia, 25 (9.1 per cent) assessed primary ventral hernia only, 119 (43.4 per cent) assessed incisional ventral hernia only, and one study (0.4 per cent) provided no information. Individual study characteristics are shown in *Table 1*.

**Table 1:** Characteristics of the 274 studies. One paper (Lambrecht et al. reported two separate studies, one RCT and one retrospective study, making the total 275)

	***n***
Centre^*^	*n*=275
Single	212 (77.1)
Multicentre	63 (23.0) (22.9)
**Study design**	*n*=275
Observational	126 (46.0) (45.8)
Prospective	26 (9.5) (9.5)
RCT	31 (10.9) (11.3)
Retrospective	92 (33.6) (33.5)
**Continent**	*n*=275
Africa	3 (1.1) (1.1)
Asia	18 (6.5) (6.5)
Australia	1 (0.4) (0.4)
Europe	115 (42.0) (41.8)
North America	137 (49.6) (49.8)
South America	1 (0.4) (0.4)
**No. of participants**	
Median (i.q.r.)	128 (77–251)
Range	21–13 567
**Prognostic factor type^†^**	*n*=386
Preoperative	136 (35.2)
Intraoperative	204 (52.8)
Postoperative	46 (11.9)
**Population**	*n*=275
Primary and incisional	129 (47.1)
Primary	25 (9.1)
Incisional	119 (43.4)
No information	1 (0.4)
**Method of detection**	*n*=275
Imaging with ultrasound or CT alone	1 (0.4)
Clinical assessment	54 (19.6)
Clinical assessment with CT	43 (15.6)
Clinical assessment with ultrasound	11 (4.0)
Clinical assessment with imaging	22 (8.0)
Clinical assessment with telephone	14 (5.1)
Clinical assessment with questionnaire	2 (0.7)
Medical records	19 (6.9)
Reoperation rate	6 (2.2)
Mixture of methods	79 (28.7)
No information	24 (8.7)
**Severe disease included**	*n*=275
Yes	198 (72.0)
No	52 (18.9)
No information	25 (9.1)
**Duration of follow-up (months)^‡^**	
Median (i.q.r.)	24 (15–39)
Range	2–116

Values in parentheses are percentages unless indicated otherwise.

* One published manuscript contained two separate studies: one multicentre and a second single-centre study.

† Studies can have a mixture of preoperative, intraoperative and postoperative prognostic factors.

‡ Five studies did not report duration of follow-up time; for studies divided by subgroups; if the difference was less than 4.5 months the mean was taken (21 studies), and if difference was more than 4.5 months the minimum length of follow-up was taken (22 studies).

Recruitment dates coincided in 109 studies that described overlapping patient cohorts (*Appendix S3*) and were excluded from meta-analysis. Some 198 studies (72.3 per cent) included both mild and severe disease; 16 (5.8 per cent) assessed severe disease only, 51 (18.6 per cent) included mild disease only, and 25 (9.1 per cent) provided no severity information.

Hernia recurrence was defined by only a minority of studies (66 of 274, 24.1 per cent), using 41 different, unstandardized definitions **(***Appendix S4***)**. Detection of hernia recurrence also varied widely, with 67 different detection methods used (*Appendix S5*). Duration of follow-up varied, with median 24 (i.q.r. 15–39; range 2–116) months.

### Risk of bias

**Fig. 2:**
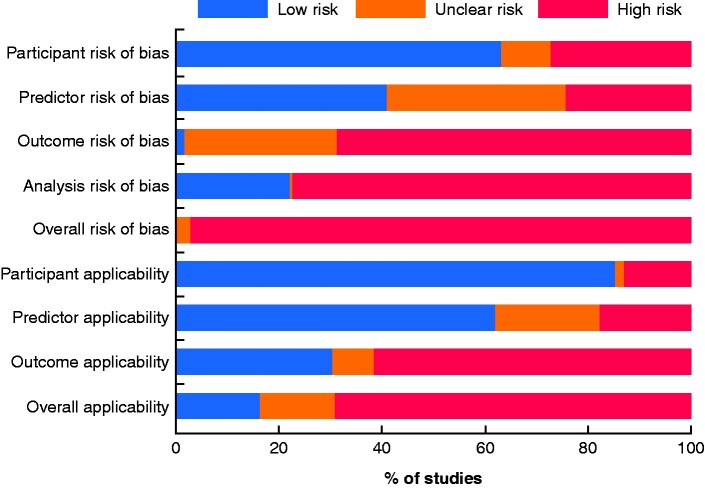
Risk-of-bias graph using an adapted version of the PROBAST^11^ The graph illustrates the present authors’ judgements for each risk-of-bias category, presented as percentages across all included studies. PROBAST, Prediction model Risk Of Bias ASsessment Tool.

Risk of bias and applicability across all studies is presented in *Fig. 2*. Most studies had high risk of bias in at least one domain, resulting in 266 studies (97.1 per cent) rating high for risk of bias overall. Eight studies (2.9 per cent) were rated unclear. No study reported acceptable data, with a low risk of bias. Notably, 272 studies (99.3 per cent) were rated as having either at high or unclear risk of bias when assessing the definition and detection of the outcome of the present study: recurrence. Concern regarding ‘overall applicability’ was rated as low in 45 studies (16.4 per cent), unclear in 40 (14.6 per cent) and high in the remaining 189 (69.0 per cent).

### Predictors of hernia recurrence

**Fig. 3:**
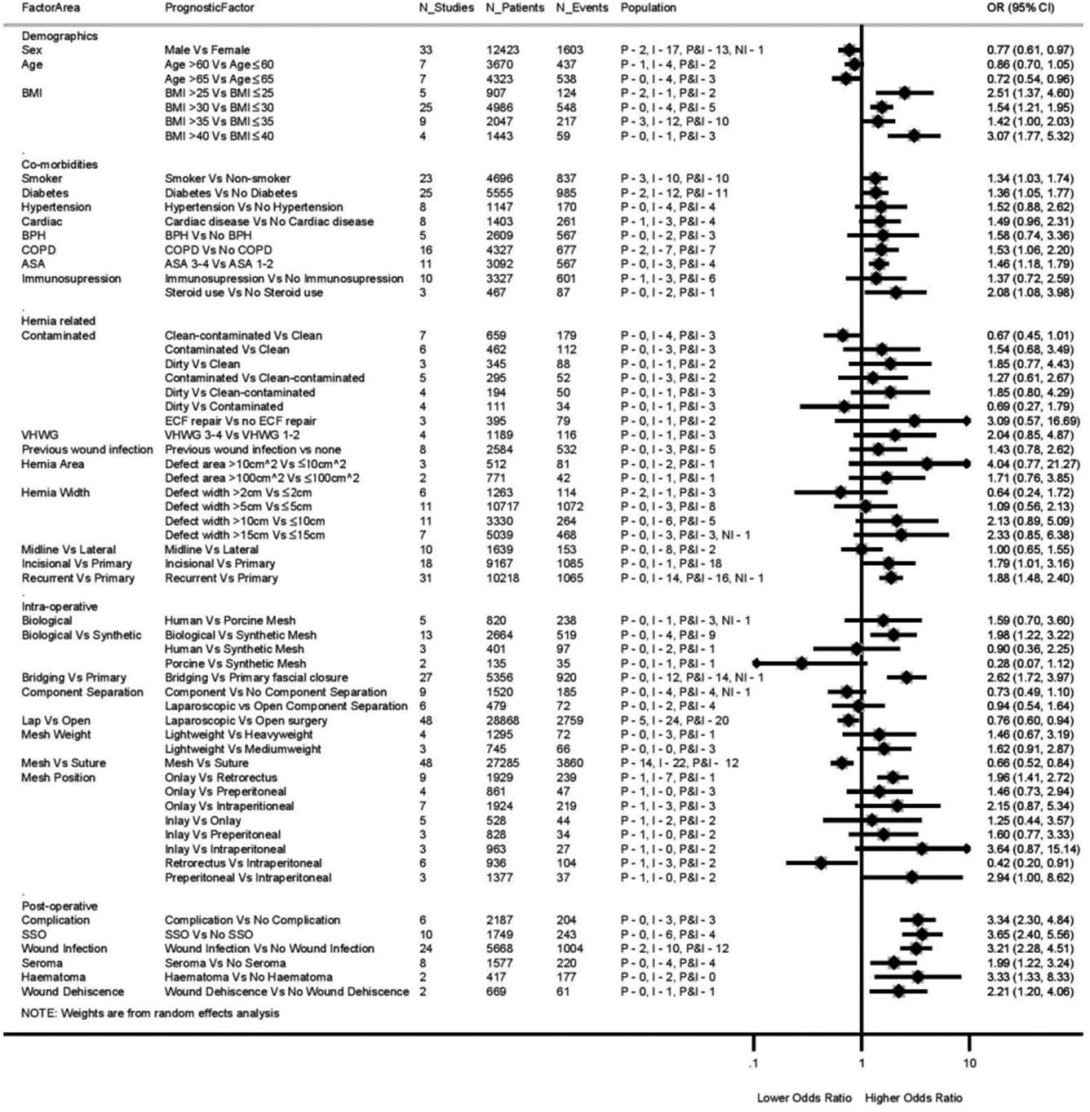
Overall meta-analysis results, showing the number of studies, patients and hernia recurrence events Values in parentheses are 95 per cent confidence intervals. Weights are from random-effects analysis. OR, odds ratio; BPH, benign prostatic hypertrophy; COPD, chronic obstructive pulmonary disease; VHWG, Ventral Hernia Working Group; SSO, surgical-site occurrence. Further details, including the population/hernia type, can be found in *Appendix S6*.

Overall, 63 individual predictors of hernia recurrence were identified: 34 preoperative (54 per cent) (16 (25 per cent) patient variables; 18 (29 per cent) hernia variables), 19 intraoperative (30 per cent), and six postoperative (10 per cent) predictors. Forty (63 per cent) of these predictors had data provided by five or more individual studies and were thus available for meta-analysis. An additional 19 predictors (30 per cent) with data from fewer than five studies, but labelled as clinically important, were also included in a meta-analysis (*Fig. 3* and *Appendix S6*). A remaining four predictors (6 per cent), with fewer than five studies providing data, were deemed clinically important enough for forest plots only (*Appendix S7*). Data were extracted for a further 172 predictors. These predictors were neither meta-analysed nor illustrated in forest plots as the data were extracted from four or fewer studies, or were insufficient to permit meta-analysis or forest plot, or the predictors were considered clinically unimportant. A list of these predictors can be found in *Appendix S8*. *Fig. 3* and *Appendix S9* present overall meta-analysis results, number of studies, patients, hernia recurrence events, and included study populations. *Appendices S6* and *S7* presents forest plots showing individual study results.

### Preoperative predictors

#### Patient predictors

Three patient factors, namely sex, age and BMI, were meta-analysed (*Fig. 3* and *Appendix S6*): for age and BMI, data were provided for different thresholds including: age above or below 60 years, age above or below 65 years, BMI above or below 25 kg/m^2^, BMI above or below 30 kg/m^2^, BMI above or below 35 kg/m^2^, BMI above or below 40 kg/m^2^. Men had significantly lower odds of recurrence (OR 0.77 (95 per cent c.i. 0.61 to 0.97); 33 studies). Both age above 60 years compared with 60 years or less and age above 65 *versus* 65 years or lessdecreased the odds of recurrence (OR 0.86 (0.70 to 1.05) and OR 0.72 (0.54 to 0.96); both 7 studies), but this was not significant or only marginally significant respectively. All BMI thresholds yielded significantly higher odds of recurrence for more obese patients (*Fig. 3* and *Appendix S6*). The BMI cut-off point was 30 kg/m^2^ in most studies; meta-analysis at this threshold gave OR 1.54 (1.21 to 1.95).

Meta-analyses of patient co-morbidities identified many factors potentially significantly associated with recurrence. Smoking, diabetes, chronic obstructive pulmonary disease (COPD), ASA grade III–IV and steroid use all had significantly higher odds of recurrence (OR 1.34, 1.36, 1.53, 1.46 and 2.08 respectively).

Meta-analysis of co-morbidity *versus* no co-morbidity for hypertension, cardiac disease, benign prostatic hypertrophy and any type of immunosuppression revealed the majority of individual study results in the direction of higher risk of recurrence, but these meta-analysis results were not statistically significant. In most studies, the recurrence rate in co-morbid patients was relatively low.

#### Hernia predictors

Two predictors relating to hernia morphology and contamination status were found to be significantly predictive of recurrence: incisional *versus* primary ventral hernia (OR 1.79 (95 per cent c.i. 1.01–3.16); 18 studies) and recurrent *versus* primary ventral hernia (OR 1.88 (1.48 to 2.40); 31 studies). Studies used a range of hernia widths to define thresholds for comparison: a wider defect appeared to predispose increasingly to recurrence, with cut-off points of 2, 5, 10 and 15 cm yielding progressively larger ORs (0.64, 1.09, 2.13 and 2.33 respectively). However, meta-analyses at these individual thresholds were not statistically significant. Hernia defect area was reported with thresholds of above or below 10 and 100 cm^2^, and gave ORs of 4.04 and 1.71 respectively; neither was significant. Hernia location (midline *versus* lateral) demonstrated no relationship with recurrence (OR 1.00 (0.65 to 1.55); 10 studies).

For most of the remaining hernia-related factors expected to be detrimental (*Fig. 3*), the meta-analysis ORs exceeded 1.00, suggesting increased risk, but results were not statistically significant as the 95 per cent c.i. spanned 1.00. Although 16 studies (5279 patients, 1018 recurrences) contributed to meta-analyses of Centres for Disease Control (CDC) wound criteria, these studies spanned six different comparisons (*Fig. 3*), so no individual meta-analysis demonstrated a significant association with recurrence; even dirty *versus* clean wounds were not significant (OR 1.85 (95 per cent c.i. 0.77 to 4.43); 3 studies).

Data on VHWG grade (3–4 *versus* 1–2) were extracted from only four studies, and meta-analysis was not significant: OR 2.04 (95 per cent c.i. 0.85 to 4.87). Previous wound infection *versus* no previous wound infection, another marker of contamination, was also not significant (OR 1.43 (0.78 to 2.62); 8 studies).

### Intraoperative predictors

Data were sufficient to meta-analyse 18 intraoperative predictors (*Fig. 3* and *Appendix S6*). These were subgrouped according to operative technique, mesh *versus* suture, mesh type, mesh weight and mesh location. For biological mesh, human *versus* porcine acellular dermal matrix (ADM) was not significant (OR 1.59 (95 per cent c.i. 0.70 to 3.60); 5 studies). Several studies provided data comparing any biological mesh with any synthetic mesh; meta-analysis suggested recurrence was significantly more frequent with biological mesh (OR 1.98 (1.22 to 3.22); 13 studies). However, data comparing biological mesh subtypes with synthetic mesh were equivocal: human ADM *versus* synthetic mesh (OR 0.90 (0.36 to 2.25); 3 studies) and porcine ADM *versus* synthetic mesh (OR 0.28 (0.07 to 1.12); 2 studies). Bridged repair was associated significantly with recurrence compared with primary fascial closure (OR 2.62 (1.72 to 3.97); 27 studies). Component *versus* no component separation did not reduce recurrence significantly (OR 0.73 (0.49 to 1.10); 9 studies), with divergent individual study results. Similarly, laparoscopic (endoscopic) component separation did not differ significantly from open component separation (OR 0.94 (0.54 to 1.64); 6 studies). Laparoscopic repair reduced the odds of recurrence significantly compared with open repair (OR 0.76 (0.60 to 0.94); 48 studies).

Regarding mesh weight, lightweight mesh did not appear to result in more recurrence than either heavyweight or medium-weight mesh (OR 1.46 (95 per cent c.i. 0.67 to 3.19), 4 studies; and OR 1.62 (0.91 to 2.87), 3 studies, respectively). Ventral hernia mesh repair *versus* suture only reduced the odds of recurrence significantly (OR 0.66 (0.52 to 0.84); 48 studies). Mesh location was significant when comparing onlay with retrorectus positions (OR 1.96 (1.41 to 2.72); 9 studies), retrorectus *versus* intraperitoneal (OR 0.42 (0.20 to 0.91); 6 studies) and preperitoneal *versus* intraperitoneal (OR 2.94 (1.00 to 8.62); 3 studies), ultimately favouring the retrorectus location significantly. Meta-analysis of other mesh locations (plane) were not significant (*Fig. 3* and *Appendix S6*).

### Postoperative predictors

Meta-analysis of postoperative predictors (*Fig. 3* and *Appendix S6*) suggested that any postoperative complication (such as pneumonia, urinary tract infection or pulmonary embolus) *versus* none increased recurrence significantly (OR 3.34 (95 per cent c.i. 2.30 to 4.84); 6 studies). Wound morbidity, defined as a surgical-site occurrence (SSO), also increased recurrence significantly (OR 3.65 (2.40 to 5.56); 10 studies). In fact, all wound complication subtypes predisposed to recurrence significantly: postoperative wound infection *versus* no infection (OR 3.21 (2.28 to 4.51); 24 studies), postoperative seroma *versus* no seroma (OR 1.99 (1.22 to 3.24); 8 studies), postoperative haematoma *versus* no haematoma (OR 3.33 (1.33 to 8.33); 2 studies) and postoperative wound dehiscence *versus* no dehiscence (OR 2.21 (1.20 to 4.06); 2 studies).

## Discussion

Over the past two decades, hernia surgeons have published a considerable volume of research describing the preoperative, intraoperative and postoperative variables that influence postoperative outcomes. This study investigated specifically how these variables influence recurrence, arguably the single most important outcome as recurrence determines whether the reconstruction was ultimately successful. Identification of predictors of recurrence is pivotal, because the decision whether to perform reconstruction or not pivots on the chance of success. However, clear signals of success are frustrated by individual primary studies that are usually relatively small, single-centre, and assess a limited handful of predictors. Moreover, small sample size bias causes divergent results that then frustrate the identification of valid predictors. Synthesizing all available evidence together by systematic review and meta-analysis, and then presenting results as forest plots, allows individual clinicians to interpret data from multiple primary studies and facilitates discussion of evidence for clinical guidelines, clinical practice, and future research. Furthermore, the strength of evidence is enhanced by excluding results for predictors with few reports (unless deemed clinically important).

The present review does have limitations, the majority contingent on the quality of primary component studies. As noted already, true prognostic research was surprisingly sparse. As anticipated, study methods were heterogeneous, and so meta-analysis had to be done across different designs, definitions of recurrence, methods for detecting recurrence, and different follow-up durations. Such variability likely underlies disparity between the study effect estimates seen across results. Because data were heterogeneous, the authors stress that interpretation of these findings should focus on factors that appeared predictive rather than on the precise strength of that prediction (whether or not the c.i. was significant). Particularly for preoperative predictors and mesh location, the small number of studies was such that findings from future larger and more rigorous studies will be important. Furthermore, extracting overall study results from the published primary literature instead of individual patient data means that multivariable analysis could not be used to account for confounding or ecological bias due to associations of multiple factors within individual patients. For example, biological mesh seems to predispose to recurrence compared with synthetic mesh, but multivariable analysis would be needed to understand whether this was independent of mesh plane. Similarly, component separation did not appear to reduce recurrence, but multivariable analysis would be needed to understand whether this result was confounded by more severe disease (in which this procedure is performed) and/or whether the repair was bridged or closed primarily.

An adapted version of the PROBAST was used to assess risk of bias^11^ and, analogous with the limitations mentioned above, it was found that most of the primary literature demonstrated high risk of bias. Predictors were usually poorly defined, and the methods and definitions used to detect recurrence also lacked standardization. In addition, blinded reporting of both predictors and hernia recurrence was unusual. In other words, it was unclear whether predictor assessment was made blinded to recurrence, or vice versa (the former being possible only in retrospective studies). This is pivotal for unbiased prognostic data^14^.

Because the aim was to evaluate the entirety of the prognostic literature, the present review was extensive, with screening of approximately 7500 abstracts. Surprisingly, very few true prognostic studies were encountered—those designed *a priori* specifically to identify predictors of ventral hernia recurrence^15–18^. Accordingly, a considerable amount of data had to be extracted from cohort studies^19–21^ that analyse how one variable (such as open *versus* laparoscopic hernia repair) affects outcome. Simple 2×2 tables could be constructed from these comparative studies and meta-analysed subsequently. Data were also extracted from large database studies^22–24^ that record the effect of multiple perioperative variables on multiple postoperative outcomes, often including hernia recurrence. There was a huge range in the amount of data extractable for individual predictors. For some commonly quoted clinical risk factors for recurrence, such as previous abdominal aortic aneurysm repair or connective tissue disorders (such as Ehlers–Danlos syndrome), there were insufficient data for meta-analysis. Analogous with this, hernia location could be categorized only into medial and lateral subgroups, as data were insufficient to identify subgroups such as suprapubic *versus* umbilical, or subxiphisternal *versus* subcostal. These subgroups are often discussed amongst hernia surgeons as ‘difficult to repair’, and with a high recurrence rate^25^. In contrast, it was possible to extract abundant data for other variables: for laparoscopic *versus* open and mesh *versus* suture repair, data were extracted from 48 studies of 28 868 and 27 285 patients respectively. Accordingly, research appears focused on potential predictors related to surgical technique and less on others that may be equally or indeed more important. Further work is required on predictors that have had limited interrogation.

This work is presented methodically in *Fig. 3*, where predictors are separated into groups: patient demographics, hernia characteristics, intraoperative and postoperative variables. BMI, smoking, diabetes, COPD, ASA grade III–IV and steroid use were found to be patient variables significantly associated with recurrence. This analysis also suggests that male sex and age above 65 years is protective. Why men should be less vulnerable to recurrence is unclear. For the age thresholds of 60 and 65 years, Kokotovic and colleagues^22^, in a publication from the Danish Ventral Hernia Database (DVHD), contributed most patients, dominating the meta-analysis. The DVHD uses reoperation rate as a surrogate for recurrence. Their large cohort suggests that elderly patients are significantly less likely than younger patients to undergo reoperation. This result is unsurprising, as elderly patients would appear less fit for a second elective repair^26^. The only hernia-related variables associated significantly with increased recurrence were incisional *versus* primary and recurrent *versus* primary ventral hernias, a finding that is well established^27^^,^^28^. In other words, previous surgical intervention causes scarring, weakens the abdominal wall, and leads to impaired wound-healing and hernia recurrence. Multiple studies have described hernia width using different thresholds, each of which appeared unassociated with recurrence. However, the present study found that as defect width increased from cut-off points of 2, 5, 10 and 15 cm, so did the odds of recurrence (OR 0.64, 1.09, 2.13 and 2.33 respectively). Even though their individual c.i. values crossed 1.00, this observation was consistent, suggesting that increasing hernia width is a genuine risk factor for recurrence. More data are required to confirm this. Furthermore, width may be measured clinically, during surgery and by imaging, all of which will be subject to interobserver and intraobserver variation, and to variation between methods. Larger defects require additional reconstructive techniques, which may, in turn, confound the predictive power of defect width. In addition, indexed publications usually arise from experienced centres, for whom larger width may be less challenging or, conversely, they attract the most difficult patients. Lastly, data on VHWG grade could be extracted from only four studies^29–32^. This scale was proposed in 2010^10^, but few publications have validated it subsequently. In the present study, no significant association was observed with recurrence when VHWG grade 3–4 was compared with grade 1–2. Similarly, CDC status was not associated with recurrence, but, again, few studies provided extractable data. Further prognostic research is required for these factors, and on creating a contamination scale that is potentially associated with postoperative outcomes.

The present analysis of intraoperative variables has confirmed the well known ‘protective’ effect of mesh over suture repair^33^, and also that primary fascial closure^34^ results in a more reliable repair than bridged; both of these associations are well established. Furthermore, the present data are consistent with biological mesh being ‘weaker’ than synthetic mesh, with greater tendency towards recurrence, an association published previously^35^^,^^36^, but perhaps less well known. In addition, laparoscopic ventral hernia repair appeared protective, again suggested by individual previous publications^6^^,^^24^, and similarly less well known and clinically accepted. In this study, mesh location was described using the ICAP (international classification of abdominal wall planes) system published recently^37^. The results concerning mesh location suggest that the retrorectus plane reduces recurrence compared with onlay and intraperitoneal planes, and that the intraperitoneal is superior to the preperitoneal plane. Observational studies, database studies and systematic reviews support this finding^24^^,^^38–41^. Lastly, the review suggests that wound morbidity (defined as SSO) leads to delayed wound-healing and subsequent recurrence. Indeed, all local wound complications were found to be associated with recurrence.

Moving forwards, prospective ventral hernia prognostic studies should be performed to eliminate bias, with well characterized participants, blinded assessment of potential predictors and outcomes, standard definitions and detection methods for both predictors and outcomes. Expert statistical support from statisticians specifically interested in prognostic research is also required to assist both design and data collection to minimize risk of bias, and so that the data generated are generalizable. If authors intend to use these prognostic data to develop prognostic models of postoperative outcomes, they should adhere to the TRIPOD (Transparent Reporting of a multivariable prediction model for Individual Prognosis Or Diagnosis) statement, a 22-item checklist that aims to improve the reporting of studies developing, validating or updating prediction models^42^^,^^43^.

*Disclosure.* A.C.J.W. declares conflicts of interest not related directly to the submitted work: consultant adviser for TELA Bio; educational grants and speaker fees for BARD, LifeCell and Cook. The authors declare no other conflict of interest.

## Funding

This work was funded by the UK National Institute for Health Research (Grant RfPB PB-PG-0816-20005) and Allergan PLC. Neither funders have been involved in the planning, methodology, analysis or write up of the research. National Institute of Health Research, Room 132, Richmond House, 79 Whitehall, London, SW1A 2NS. Allergan Plc, Clonshaugh Business and Technology Park, Coolock, Dublin, D17 E400, Ireland. Mallett S was supported by NIHR Birmingham Biomedical Research Centre at the University Hospitals Birmingham NHS Foundation Trust and the University of Birmingham. Halligan S is also supported by the NIHR University College London Hospitals Biomedical Research Centre. This report presents independent research supported by the National Institute for Health Research (NIHR). The views and opinions expressed by authors in this publication are those of the authors and do not necessarily reflect those of the NHS, the NIHR, or the Department of Health.

*Disclosure.* A.C.J.W. declares conflicts of interest not related directly to the submitted work: consultant adviser for TELA Bio; educational grants and speaker fees for BARD, LifeCell and Cook. The authors declare no other conflict of interest.

## Supplementary material

Supplementary material is available at *BJS Open* online.

## Supplementary Material

zraa071_Supplementary_Data
