# Corrigendum to: Identifying predictors of ventral hernia recurrence: systematic review and meta-analysis

**DOI:** 10.1093/bjsopen/zrab047

**Published:** 2021-06-16

**Authors:** S G Parker, S Mallett, L Quinn, C P J Wood, R W Boulton, S Jamshaid, M Erotocritou, S Gowda, W Collier, A A O Plumb, A C J Windsor, L Archer, S Halligan

*BJS Open*, 2021, DOI: 10.1093/bjsopen/zraa071

The following funder information was omitted from this article upon first publication but has now been added:

This work was funded by the UK National Institute for Health Research (Grant RfPB PB-PG-0816-20005) and Allergan PLC. Neither funder was involved in the planning, methodology, analysis or write-up of the research. National Institute of Health Research, Room 132, Richmond House, 79 Whitehall, London, SW1A 2NS. Allergan Plc, Clonshaugh Business and Technology Park, Coolock, Dublin, D17 E400, Ireland.

S.M. was supported by NIHR Birmingham Biomedical Research Centre at the University Hospitals Birmingham NHS Foundation Trust and the University of Birmingham during this work. S.H. is supported by the NIHR University College London Hospitals Biomedical Research Centre.

This report presents independent research supported by the National Institute for Health Research (NIHR). The views and opinions expressed by authors in this publication are those of the authors and do not necessarily reflect those of the NHS, the NIHR, or the Department of Health.

