# Supplementary material for: Identifying predictors of ventral hernia recurrence: systematic review and meta-analysis
Source: BJS Open. 2021 Apr 11;5(2):zraa071. doi: 10.1093/bjsopen/zraa071 (PMC8038271; doi:10.1093/bjsopen/zraa071)
Supplement: zraa071_Supplementary_Data [file zraa071_supplementary_data.zip › OnlineResource1.docx]

**Online Resource 1**

**General Search**

((((((((("General Surgery"[MESH]) OR "Reconstructive Surgical Procedures"[MESH]))

OR ((("pneumoperitoneum"[Title/Abstract]) OR "botox"[Title/Abstract]) OR

"botulinium"[Title/Abstract])) OR (((("two stage"[Title/Abstract]) OR "stage

repair"[Title/Abstract]) OR "staged repair"[Title/Abstract]) OR "two step"[Title/Abstract])) OR ((("component separation"[Title/Abstract]) OR "transversus

abdominis"[Title/Abstract]) OR "retrorectus"[Title/Abstract])) OR (((("bridging"[Title/Abstract]) OR "bridge repair"[Title/Abstract]) OR "bridged

repair"[Title/Abstract]) OR "silo"[Title/Abstract])) OR (("open"[Title/Abstract]) OR

"laparoscopic"[Title/Abstract]))) AND (((((((hernia[Title/Abstract]) OR "abdominal wall defect"[Title/Abstract]) OR "abdominal wall reconstruction"[Title/Abstract]) OR "ventral defect"[Title/Abstract]) OR "enterocutaneous fistula"[Title/Abstract])) OR ("Hernia"[Mesh] OR "Hernia, Abdominal"[Mesh] OR "Hernia, Ventral"[Mesh] OR "Hernia, Umbilical"[Mesh]))

Filters: Publication date from 1995/01/01 to 2017/12/31; Humans; English; Adult: 19+

Years

**Prognostic/Predictive studies search**

((((ventral hernia[MeSH Terms]) OR abdominal hernia[MeSH Terms])) AND (((predictive[Title/Abstract]) OR predictor[Title/Abstract]) OR factor[Title/Abstract])) AND ((recurrence[Title/Abstract]) OR recurrent hernia[Title/Abstract])

Filters: Publication date from 1995/01/01 to 2017/12/31; Humans; English; Adult: 19+ Years
