# Supplementary material for: Identifying predictors of ventral hernia recurrence: systematic review and meta-analysis
Source: BJS Open. 2021 Apr 11;5(2):zraa071. doi: 10.1093/bjsopen/zraa071 (PMC8038271; doi:10.1093/bjsopen/zraa071)
Supplement: zraa071_Supplementary_Data [file zraa071_supplementary_data.zip › OnlineResource2.PROBAST.docx]

## Online Resource 2

## Risk of Bias (ROB) criteria based on PROBAST.

**Possible answers: Yes (Y), probably yes (PY), probably no (PN), No (N), or No information (NI).**

| **Participants** | | |
| --- | --- | --- |
| **Risk of Bias** | | |
| 1.1: Were appropriate data sources used, e.g. cohort, RCT or nested case-control study data? | HIGH risk: Case-control studies  Low risk: RCTs, cohort studies, database studies | |
| 1.2: Were all inclusions and exclusions of participants appropriate? | High risk: Studies including some emergency repairs some paediatric hernia repairs (only if participants were 10% emergency repairs, 10% paediatric repairs were the studies included), studies with primary hernias only.  Low risk: Studies of elective ventral hernia repair (incisional only, and incisional & primary VH) | |
| 1.3: Were patients with severe disease included in analysis? | High risk: Severe disease defined as ventral hernias with a diameter of >10cm, or with active contamination (VHWG grade 4). If studies only included ventral hernias with severe disease then defined as high risk.  Low risk: Studies including ALL ventral hernias (as our prognostic model wants to applied to ALL VHs) | |
| **Risk of bias introduced by selection of participants (low, high, unclear)** | **High: Any of the domains answer is N or PN**  **Unclear: All domains answer Y or PY or NI**  **Low: All domains answered Y or PY** | |
| **Applicability** |  | |
| Concern that the included participants and setting do not match the review question | High: Demographics or comorbidity limitations for study participants (e.g. diabetics, sex etc). If study is primary ventral hernia only, or contains 10% either emergency or paediatrics hernia repairs. If study contains hernias <5cm in diameter only.  Unclear: If inclusion criteria are ‘unclear’  Low: If >5cm diameter incisional ventral hernia with or without primary ventral hernia | |
| **Predictors** | | |
| **Risk of Bias** | | |
| 2.1: Were predictors defined and assessed in a similar way for all participants? | | High risk: Patient questionnaire, telephone consultation, non-clinician collecting predictor data (including filling database information), if multiple methods used.  Low risk: Medical records, clinical notes, medical database, clinical review, imaging |
| 2.2: Were predictor assessments made without knowledge of outcome data? | | High risk: Retrospective data collection outcome data is likely to be present. Some predictors open to bias if outcome data known eg Predictors such as hernia characteristics (contamination status, width, LoD, wound infection/events).  Low risk: Prospective data collection, Prospective maintained database – retrospectively reviewed – benefit of the doubt |
| **Risk of bias introduced by predictors or their assessment** | | **High: Any of the domains answer is N or PN**  **Unclear: All domains answer Y or PY or NI**  **Low: All domains answered Y or PY** |
| **Applicability** | |  |
| Concern that the definition, assessment or timing of predictors in the model do not match the review question | | High: Concern may be raised if a predictor was measured/detected outside standard clinical practice for example: smoking status using urine cotinine, COPD diagnosis using spirometry etc…  Unclear: If there was little information about how predictors were assessed and defined and predictors were not discrete.  Low: Our review question is relatively generalised with few specifics on predictor requirements eg. detection methods or definitions or timing of predictor evaluation. |
| **Outcomes** | | |
| **Risk of Bias** | | |
| 3.1: Was the outcome determined appropriately? | | High risk: Patient questionnaire, telephone questionnaire, medical records, and re-operation rate  Low risk: Clinical detection of hernia recurrence e.g. clinical examination, US scan and CT |
| 3.2: Was a pre-specified or standard outcome definition used? | | High risk: If there is reason to suspect that a definition has been chosen so the study gets a positive results  Low risk: If a pre-specified or standardised definition is used  No information: No definition |
| 3.3: Were predictors excluded from the outcome definition? | | High risk: If recurrence definition includes a predictor variable  Low risk: Likely to be low for all studies; currently no definition that includes a predictor variable |
| 3.4: Was the outcome defined and determined in a similar way for all participants? | | High risk: If multiple different methods used to detect the outcome (e.g. telephone and questionnaire). If multiple different reporters (non-consultants) detect recurrence (outcome)  LOW risk: Same detection method for recurrence for all participants |
| 3.5: Was the outcome determined without knowledge of predictor information? | | High risk: In prospective trials where the outpatient assessor was unblinded, retrospective data collection where knowledge of the outcome is likely to be present.  Low risk: Prospective trials with blinded outpatient assessment; includes independent blinded radiological assessment. Trials with no difference in abdominal scarring. |
| 3.6: Was the time interval between predictor assessment and outcome determination appropriate? | | High risk: Follow-up of less than 6 months, difference in follow-up in treatment arms of >4.5 months.  Low risk: Follow-up of ≥6 months. |
| **Risk of bias introduced by the outcome or its determination** | | **High: Any of the domains answer is N or PN**  **Unclear: All domains answer Y or PY or NI**  **Low: All domains answered Y or PY** |
| **Applicability** | |  |
| Concern that the outcome, its definition, timing or determination do not match the review question | | High: Some studies may include bulging in their definition for recurrence. Bulging has no abdominal wall defect and therefore is outside the definition for recurrence.  Unclear: No information about how recurrence was defined or detected.  Low: Our review question includes all different definitions for recurrence that imply there is an abdominal wall defect. |
| **Analysis** | | |
| **Risk of Bias** | | |
| 4.1: Were there a reasonable number of participants with the outcome? >10 events | | High risk: <10 events per variable (if multivariate analysis performed). This will be the case from most studies.  Low risk: ≥10 events per variable (if multivariate analysis performed), if univariate analysis performed only 10 events required. |
| 4.2: Were all enrolled participants included in the analysis? | | High risk: >10% of participants not included due to loss to follow up or another systematic reason (like missing data).  Low risk: If this is <10% and there appears to be no selection or inclusion criteria for the final analysis |
| 4.3: Were participants with missing data handled appropriately? | | Not rated as not reported in any articles |
| 4.4: Did they report estimates from at least 3 standard clinical variables, or justify why not? | | High risk: Less than 3 of the standard clinical variables below reported*  Low risk: At least 3 of the standard clinical variables below reported* |
| 4.5: Were at least 3 predictor estimates reported with non-statistically significant results? | | High risk: ≤2 predictor estimates with no statistical  Low risk: ≥3 predictor estimates with no statistical significance |
| **Risk of bias introduced by the analysis** | | **High: Any of the domains answer is N or PN**  **Unclear: All domains answer Y or PY or NI**  **Low: All domains answered Y or PY** |
| **Overall judgement** | | |
| **Overall judgement of risk of bias** | | **HIGH if risk of bias for any domain is HIGH**  **LOW if risk of bias for all domains is LOW**  **UNCLEAR if risk of bias for one or more domains is UNCLEAR and the other domains are all rated as LOW** |
| **Overall judgement of applicability** | | **HIGH if concern for applicability for any domain is HIGH**  **LOW if concern for applicability for all domains is LOW**  **UNCLEAR if concern for applicability one or more domains is UNCLEAR and the other domains are all rated as low** |

*Standard pre-operative clinical variables that should be reported:

| Age |
| --- |
| BMI |
| COPD |
| Smoker |
| Diabetes |
| Primary hernia or Incisional hernia |
| Previous hernia repair |
| Hernia defect size area/width |
