# Supplementary material for: Identifying predictors of ventral hernia recurrence: systematic review and meta-analysis
Source: BJS Open. 2021 Apr 11;5(2):zraa071. doi: 10.1093/bjsopen/zraa071 (PMC8038271; doi:10.1093/bjsopen/zraa071)
Supplement: zraa071_Supplementary_Data [file zraa071_supplementary_data.zip › OnlineResource3.Recruitment.docx]

Online Resource 3 – Recruitment

| **Study characteristics** |  |  |  |
| --- | --- | --- | --- |
|  |  |  |  |
| **Study** |  | **Recruitment** |  |
| **Name/year** | **Country** | **Dates** | **Notes** |
| **Sadava 2016** | Argentina |  |  |
| **Pring 2008** | Australia |  |  |
| **Werkgartner 2014** | Austria | Nil |  |
| **Kohler 2015a** | Austria | Jan 2009 to Oct 2013 | Primary - no overlap |
| **Kohler 2015b** | Austria | 2009 to 2013 | Incisional - no overlap |
| **Muysoms 2013** | Belgium |  |  |
| **Berrevoet 2011** | Belgium | Feb 2004 to Apr 2007 | Primary - no overlap |
| **Berrevoet 2010** | Belgium | March 2000 to Apr 2006 | Incisional - no overlap |
| **Bontinck 2014** | Belgium | Apr 2009 to Dec 2011 |  |
| **Lahon 2009** | Belgium | Jan 2003 to Feb 2007 |  |
| **Hauters 2017** | Belgium | 2001 to 2014 |  |
| **Chelala 2016** | Belgium | Oct 2000 to Mar 2014 |  |
| **Tollens 2011** | Belgum | May 2004 to Feb 2009 |  |
| **Park 1998** | Canada |  |  |
| **Dinh Le 2013** | Canada | Apr 2008 to Dec 2011 |  |
| **Birch 2007** | Canada | Aug 1999 to Jun 2004 |  |
| **Chan 2005** | Canada | Jan 1999 to Dec 2000 |  |
| **Han 2007** | China |  |  |
| **Vidovic 2006** | Croatia |  |  |
| **Christoffersen 2015a** | Denmark | Jan 2008 to Jan 2012 | LIKELY OVERLAP WITH **HELGSTRAND 2013** |
| **Christoffersen 2015b** | Denmark | Jan 2008 to Dec 2010 | LIKELY OVERLAP WITH **CHRISTOPHERSEN 2013** |
| **Westen 2014** | Denmark | Jan 2000 to Dec 2004 |  |
| **Christoffersen 2013** | Denmark | Jan 2007 to Dec 2010 | LIKELY OVERLAP WITH **CHRISTOPHERSEN 2015b** |
| **Helgstrand 2013** | Denmark | Jan 2007 to Dec 2010 | LIKELY OVERLAP WITH **CHRISTOPHERSEN 2015a** |
| **Oma 2017** | Denmark | Jan 2007 to Apr 2013 | LIKELY OVERLAP WITH **CHRISTOPHERSEN 2015a, 2015b, 2013,** and **HELGSTRAND 2013** but ONLY FEMALES included |
| **Kokotovic 2016** | Denmark | Jan 2007 to Dec 2010 | OVERLAP WITH **HELGSTRAND 2013** BUT **LONGER FOLLOW UP** |
| **Bessa 2015** | Egypt |  |  |
| **Afifi 2005** | Egypt |  |  |
| **Youssef 2007** | Egypt |  |  |
| **Bensaadi 2014** | France | Jan 2007 to Aug 2011 |  |
| **Luc 2014** | France | Jan 2004 to Dec 2012 |  |
| **Romain 2016** | France | Jan 2010 to May 2013 | Two different hospitals same university - no overlap |
| **Mercoli 2017** | France | 2005 to 2014 | Two different hospitals same university - no overlap |
| **Bageacu 2002** | France | Jan 1993 to Dec 1998 |  |
| **Aura 2002** | France | Jul 1994 to Oct 2001 |  |
| **Renard 2017** | France | Sep 2007 to Apr 2013 |  |
| **Demetrashvili 2017** | Georgia |  |  |
| **Korenkov 2002** | Germany | 1997 to 1999 | SAME DATASET AS **SAUERLAND 2004** |
| **Conze 2005** | Germany | Jun 1999 to Dec 2000 |  |
| **Schmidbauer 2005** | Germany | Jan 1996 to Dec 2001 |  |
| **Meyer 2015** | Germany | Jan 2006 to Jan 2011 |  |
| **Scheuerlein 2011** | Germany | Jan 2008 to July 2010 |  |
| **Wolter 2009** | Germany | Jun 2004 to June 2006 |  |
| **Langer 2005a** | Germany | 1976 to 2001 | SAME DATASET AS **LANGER 2005b** |
| **Sauerland 2005** | Germany | 1990 to 1999 |  |
| **Dietz 2014** | Germany | Jan 1999 to Dec 2006 |  |
| **Sauerland 2004** | Germany | 1997 to 1999 | SAME DATASET AS **KORENKOV 2002** |
| **Lauscher 2013** | Germany | Dec 2006 to Apr 2009 |  |
| **Langer 2005b** | Germany | 1976 to 2001 | SAME DATASET AS **LANGER 2005b** |
| **Zografos 2007** | Greece | Jan 1997 to Dec 2004 |  |
| **Tsimoyiannis 2008** | Greece | May 1996 to Dec 2005 |  |
| **Pavlakis 2006** | Greece | 1990 to Mar 2004 |  |
| **Misra 2006** | India | Apr 2003 to Apr 2005 |  |
| **Bansal 2012** | India | May 2007 to Dec 2011 | OVERLAP WITH **BANSAL 2011** |
| **Bansal 2011** | India | Apr 2008 to Mar 2010 | OVERLAP WITH **BANSAL 2012** |
| **Qadri 2010** | India | Dec 2005 to Dec 2009 |  |
| **Shukla 2005** | India | Jan 1991 to August 2003 |  |
| **Prasad 2011** | India | Jan 2005 to Dec 2009 |  |
| **Sharma 2011** | India | Jan 1992 to Jun 2005 |  |
| **Notash 2007** | Iran |  |  |
| **Froylich 2016** | Israel |  |  |
| **Olmi 2005** | Italy | Sep 2001 to May 2003 | OVERLAP WITH **OLMI 2007** |
| **Olmi 2007** | Italy | Sep 2001 to Dec 2004 | OVERLAP WITH **OLMI 2005** |
| **Navarra 2007** | Italy | Sep 2003 to Jan 2010 |  |
| **Ammaturo 2005** | Italy | Jun 2002 to ? |  |
| **Stabilini 2009** | Italy | Mar 1995 to Dec 2005 |  |
| **Cavallaro 2013** | Italy | no dates | Different Hosp from Brescia |
| **Bencini 2003** | Italy | Jan 2000 to Jun 2002 | SOME OVERLAP WITH **BENCINI 2009** (BUT **BENCINI 2003** INCLUDES 49 OPEN REPAIRS WITH 42 LAP, **BENCINI 2009** IS JUST 146 LAP REPAIRS) |
| **Brescia 2016** | Italy | Jan 2013 to Dec 2015 | Different Hosp from Cavallaro |
| **Caruso 2017** | Italy | Jan 2001 to Dec 2014 |  |
| **Asti 2016** | Italy | Sep 2001 to Dec 2014 |  |
| **Soliani 2017** | Italy | May 2004 to Jul 2014 |  |
| **Ferrarese 2016** | Italy | Mar 2008 to Mar 2014 |  |
| **Bencini 2009** | Italy | Jan 2000 to Dec 2006 | SOME OVERLAP WITH **BENCINI 2003** (BUT **BENCINI 2003** INCLUDES 49 OPEN REPAIRS WITH 42 LAP, **BENCINI 2009** IS JUST 146 LAP REPAIRS) |
| **Baccari 2013** | Italy | Feb 2003 to Dec 2010 |  |
| **Ferrai 2013** | Italy | Jan 2002 to Nov 2011 |  |
| **Tsuruta 2014** | Japan |  |  |
| **Venclauskas 2010** | Lithuania | 2005 to 2008 | Different years no overlap |
| **Venclauskas 2007** | Lithuania | 1997 to 2000 | Different years no overlap |
| **Eker 2013** | Netherlands | May 1999 to Dec 2006 | Possible overlap with Van't Riet 2002 but only for 7 months recruitment out of 7 years - therefore … no overlap |
| **Luijendijk 2000** | Netherlands | Mar 1992 to Feb 1998 |  |
| **Wassenaar 2010** | Netherlands | Aug 2005 to Jul 2008 | POSSIBLY A SMALL OVERLAP WITH **STIRLER 2014** - SAME AUTHORS AND LIKELY SAME LOCATION, BUT ONLY A FRACTION OF THE PATIENTS, ALSO OVERLAP WITH **WASSENAAR 2009** FOR 17 MONTHS |
| **Stirler 2014** | Netherlands | Jan 2000 to Sep 2012 | POSSIBLY A SMALL OVERLAP WITH **WASSENAAR 2010**, BUT ONLY A FRACTION OF THE PATIENTS, OVERLAP WITH **WASSENAAR 2009** |
| **Halm 2005** | Netherlands | Jan 1998 to Dec 2002 |  |
| **Van't Riet 2002** | Netherlands | Jan 1996 to Jan 2000 | Possible overlap with Eker 2013 but only for 7 months recruitment out of 4 years - therefore … no overlap |
| **Wassenaar 2009** | Netherlands | Jan 2001 to Dec 2007 | 100% OVERLAP WITH **STIRLER 2014,** 17 MONTHS OVERLAP WITH **WASSENAAR 2010** |
| **Slater 2015a** | Netherlands | 2000 to 2010 | OVERLAP WITH **SLATER 2015c** |
| **Slater 2015b** | Netherlands | 2000 to 2009 |  |
| **Slater 2015c** | Netherlands | Sep 2000 to Mar 2013 | OVERLAP WITH **SLATER 2015a** |
| **Vrijland 2000** | Netherlands | Sep 1982 to Aug 1998 | Possible overlap with Van't Riet 2002 but only for 28 months for 16 years recruiment (192 months). |
| **Atema 2017** | Netherlands | Jan 2011 to Feb 2015 |  |
| **Mommers 2017** | Netherlands | 2000 to 2012 |  |
| **Lambrecht 2014** | Norway | 2007 to 2010 | Same IH cohort as Lambrecht 2015, but tecting for different variables on datasheet so no overlap |
| **Gronvold 2012** | Norway | Mar 2008 to Jun 2010 |  |
| **Lambrecht 2015a** | Norway | 2007 to 2010 | Same IH cohort as Lambrecht 2014, but tecting for different variables on datasheet so no overlap |
| **Lambrecht 2015b** | Norway | Oct 2002 to Jun 2006 | No overlap |
| **Malik 2015** | Pakistan | Jun 2011 to Jun 2013 | No overlap |
| **Lal 2012** | Pakistan | Jan 2008 to Dec 2010 | No overlap |
| **Malik 2008** | Pakistan | Jan 2000 to Dec 2004 | No overlap |
| **Memon 2013** | Pakistan | Jan 2001 to Jun 2009 | No overlap |
| **Pawlak 2016** | Poland | Nov 2012 ro Aug 2013 |  |
| **Mitura 2017** | Poland | May 2011 to Jun 2014 |  |
| **Al-Salamah 2006** | Saudi Arabia |  |  |
| **Stojiljkovic 2013** | Serbia |  |  |
| **Lomanto 2005** | Singapore |  |  |
| **Asencio 2009** | Spain | no dates |  |
| **Carbajo 1999** | Spain | Jan 1994 to Jan 1997 |  |
| **Arroyo 2001** | Spain | Jun 1992 to Jan 1998 | OVERLAP WITH **ARROYO 2002** |
| **Moreno-Egea 2016** | Spain | Jan 2012 to Dec 2014 |  |
| **Moreno-Egea 2007** | Spain | Jan 1996 to Dec 2006 | OVERLAP WITH **MORENO-EGEA 2012** - ONLY 18 LUMBAR HERNIAS - - PROBABLY CAN IGNORE, OVERLAP WITH **MORENO-EGEA 2012a** - ALL HERNIAS IN THAT STUDY |
| **Arteaga-Gonzalez 2010** | Spain | Jan 2005 to Oct 2008 |  |
| **Moreno-Egea 2012** | Spain | Jan 1995 to Dec 2008 | OVERLAP WITH **MORENO-EGEA 2007** - ONLY 18/55 LUMBAR HERNIAS - PROBABLY CAN IGNORE |
| **Moreno-Egea 2012a** | Spain | Jan 1994 to Jan 2008 | OVERLAP WITH **MORENO-EGEA 2012** - 33/73 NON MIDLINE HERNIAS ARE LUMBAR AND TREATED WITH LAP SURGERY, OVERLAP WITH MORENO-EGEA 2007 - 43/73 HERNIAS IN MORENO-EGEA 2007 |
| **Martinez 2017** | Spain | Jan 2009 to Dec 2014 |  |
| **Lorente-Herce 2015** | Spain | Jan 2000 to Dec 2011 |  |
| **Porrero 2015** | Spain | Jun 2004 to Dec 2010 |  |
| **Moreno-Egea 2012b** | Spain | Jan 1994 to Jun 2009 | OVERLAP WITH **MORENO-EGEA** **2007, 2012, 2012a** |
| **Arroyo 2002** | Spain | Jun 1992 to Jan 1998 | OVERLAP WITH **ARROYO 2001** |
| **Rogmark 2016** | Sweden | no dates |  |
| **Dalenback 2013** | Sweden |  |  |
| **Israelsson 2006** | Sweden |  |  |
| **Beldi 2011** | Switzerland | Apr 2005 to Jan 2008 | 36 PATIENTS FROM HERE MAY WELL BE IN **KURMANN 2011** |
| **Kurmann 2011** | Switzerland | Feb 2003 to Jun 2009 | MAY WELL CONTAIN THE 36 PATIENTS FROM **BELDI 2011**. Possible overlap with Kurmann 2010, but I think this is a separate cohort |
| **Kurmann 2010** | Switzerland | 1994 to 2008 | Liver transplant pateints only |
| **Huang 2013** | Taiwan |  |  |
| **Barbaros 2007** | Turkey | Jan 2001 to Oct 2005 |  |
| **Polat 2005** | Turkey | Jan 2000 to Oct 2003 |  |
| **Gecim 1996** | Turkey |  |  |
| **Basoglu 2004** | Turkey | Jan 1986 to Nov 2000 |  |
| **Khan 2012** | UK | Jan 2004 to Jul 2008 |  |
| **Solomon 2010** | UK | May 1998 to Dec 2008 | No overlap with Sturt 2011 as this is primary only |
| **Ching 2008** | UK | Dec 2002 to Aug 2007 |  |
| **Sanjay 2005** | UK | no dates |  |
| **Shaikh 2013** | UK | Jan 2007 to June 2009 |  |
| **Tandon 2016** | UK | Jan 2008 to Dec 2010 |  |
| **Mann 2015** | UK | Apr 2007 to Sep 2012 |  |
| **Hornby 2015** | UK | Jan 2004 to Dec 2010 |  |
| **Giordano 2015.1&2** | UK | no dates |  |
| **Jamal 2015** | UK | 2005 to 2012 |  |
| **Shipworth 2014** | UK | Feb 2009 to Sep 2012 |  |
| **Sturt 2011** | UK | 1994 to 2008 | No overlap with Solomon 2010 as this is incisional only |
| **Light 2016** | UK | 2012 to 2015 |  |
| **Warwick 2016** | UK | Feb 2007 to Nov 2013 | Possible small overlap with Giordano 2015.1&2 but no dates for giordano and Exeter only one of 7 centres so probably negligible |
| **Itani 2010** | USA | Feb 2004 to Jan 2007 |  |
| **Wormer 2016** | USA | Oct 2012 to Feb 2015 |  |
| **Novitsky 2006** | USA | Jul 1998 to Dec 2003 | OVERLAP WITH **COBB 2006** |
| **Cobb 2006** | USA | Jul 1998 to Dec 2003 | OVERLAP WITH **NOVITSKY 2006** |
| **Heniford 2003** | USA | Nov 1993 to Feb 2003 | Multicentre probably cotains patients for Cobb 2006 and Novitsky 2006, difficult to know |
| **Cox 2016** | USA | 2007-2011 | OVERLAP - KLIMA 2014, HUNTINGTON 2016 BUT DIFFERENT PREDICTORS SO NIL CONCERNS |
| **Klima 2014** | USA | Sep 2005 to Jul 2010 | OVERLAP - COX 2016, HUNTINGTON 2016 BUT DIFFERENT PREDICTORS SO NIL CONCERNS |
| **Huntington 2016** | USA | 2005–2014 | OVERLAP - COX 2016, KLIMA 2014 BUT DIFFERENT PREDICTORS SO NIL CONCERNS |
| **Greenstein 2008** | USA | Sep 2004 to Dec 2005 |  |
| **Bingener 2007** | USA | Oct 1995 to Dec 2005 |  |
| **Bochicchio 2013** | USA | Feb 2008 to Jan 2010 |  |
| **De Maria 2000** | USA | Jan 1996 to Jun 1997 |  |
| **Nguyen 2016** | USA | Mar 2012 to Jun 2014 |  |
| **Le Blanc 2003** | USA | Jul 1992 to May 2000 |  |
| **Ng 2015** | USA | Jan 2009 to Jul 2013 |  |
| **Carbonell 2013** | USA | Aug 2007 to Feb 2013 | OVERLAP WITH **COBB 2015, WARREN 2017**, Some overlap with Warren 2015 but looking at different predictors so not a problem |
| **Cobb 2015** | USA | Aug 2006 to Aug 2013 | OVERLAP WITH **CARBONELL 2013, WARREN 2017**. Some overlap with Warren 2015 but looking at different predictors so not a problem |
| **Warren 2015** | USA | Jul 2006 to Jul 2014 | Some overlap with Warren 2017, Cobb 2015, and Carbonell 2013 - but looking at different predictors so not a problem |
| **Warren 2017** | USA | Mar 2006 to Jan 2013 | OVERLAP WITH **COBB 2015, CARBONELL 2013**, Some overlap with Warren 2015 but looking at different predictors so not a problem |
| **Iacco 2014** | USA | Jan 2007 to Jun 2011 |  |
| **Azoury 2014a** | USA | Oct 2010 to Jul 2013 | OVERLAP THE 42 **AZOURY 2014a** PATIENTS ARE IN **AZOURY 2014b**. DIFFERENT PREDICTORS FROM AZAR 2017 |
| **Azoury 2014b** | USA | Oct 2010 to Jul 2013 | OVERLAP THE 42 **AZOURY 2014a** PATIENTS ARE IN **AZOURY 2014b**. DIFFERENT PREDICTORS FROM AZAR 2017 |
| **Azar 2017** | USA | 2008 to 2015. | SOME OVERLAP WITH **AZOURY 2014a, AZOURY 2014b** BUT DIFFERENT PREDICTORS SO NO WORRIES. |
| **Berger 2014** | USA | Jan 2000 to Dec 2010 |  |
| **Richmond 2014** | USA | Jan 2006 to Dec 2012 |  |
| **Fischer 2014** | USA | 2007 to 2012 | OVERLAP WITH **WINK 2014**. No overlap with Basta 2015 as primary fascial closure only |
| **Basta 2015** | USA | Jan 2007 to Jan 2013 | OVERLAP WITH **WINK 2014**. No overlap with Fischer 2014 as bridging mesh only |
| **Wink 2014** | USA | 2007–2012 | SOME OVERLAP WITH **BASTA 2015, FISCHER 2014** |
| **Keating 2016** | USA | Dec 2009 and Jan 2013 | DIFFERENT PATIENT SET DIFFERENT SENIOR AUTHOR SERIES |
| **Cheng 2014** | USA | 2007 to 2013 |  |
| **Harth 2010** | USA | Jan 2005 to Feb 2009 | OVERLAP WITH **HARTH 2011, KRPATA 2012, ROSEN 2013, KANTERS 2012** |
| **Harth 2011** | USA | 2007 to 2010 | OVERLAP WITH **HARTH 2010, KRPATA 2012, ROSEN 2013, KANTERS 2012** |
| **Petro 2015** | USA | Patients from 2011 | OVERLAP WITH **KRPATA 2012 (SMALL), ROSEN 2013, KANTERS 2012** |
| **Krpata 2012** | USA | Mar 2006 to Mar 2011 | OVERLAP WITH **HARTH 2010, HARTH 2011, ROSEN 2013, KANTERS 2012, PETRO 2015** |
| **Rosen 2013** | USA | Sep 2005 to Feb 2012 | OVERLAP WITH **HARTH 2010, HARTH 2011, KRPATA 2012, KANTERS 2012, PETRO 2015** |
| **Kanters 2012** | USA | Mar 2006 to Jan 2012 | OVERLAP WITH **HARTH 2010, HARTH 2011, KRPATA 2012, ROSEN 2013, PETRO 2015** |
| **Rosen 2009** | USA | Dec 2005 to Apr 2008 | One predictor - not in others |
| **Petro 2016** | USA | Jan 2006 to Jun 2013 | OVERLAP WITH **FAYEZIZADEH 2016**. SOME OVERLAP WITH **ROSEN2013, KANTERS 2012** (Larger cohort - some ROSEN patients but also other |
| **Fayezizadeh 2016** | USA | 2007 and 2014 | OVERLAP WITH **PETRO 2016**. Not Rosen - Novitsky |
| **Majumder 2016** | USA | Jun 2009 to Mar 2015 | Small overlap on dates with Harth 2010 and krpata 2012 but this is multicenter and contaminated probably very few if any overlap |
| **Rosen 2012** | USA | no dates | OVERLAP WITH **ITANI 2012,** (THIS STUDY IS AN INTERIM ANALSIS OF ITANI 2012 WITH LESS PATIENTS AND LESS FOLLOW UP TIME) |
| **Jin 2007** | USA | Jan 2004 to Dec 2005 | I don't think this is an 11 month overlap with Harth 2010, Rosen not main author and not single surgeon |
| **Brahmbhatt 2014** | USA | Jan 2000 to Dec 2010 | OVERLAP WITH **SUBRAMANIAN 2013, CARTER 2014** (BOTH CONTAIN LAP ONLY REPAIRS) |
| **Carter 2014** | USA | Jan 2000 to Dec 2010 | OVERLAP WITH **SUBRAMANIAN 2013, BRAHMBHATT 2014** (BOTH CONTAIN LAP ONLY REPAIRS) |
| **Subramanian 2013** | USA | 2000 to 2010 | OVERLAP WITH **BRAHMBHATT 2014, CARTER 2014** (BOTH CONTAIN LAP ONLY REPAIRS) |
| **Liang 2013** | USA | 2000 to 2010 | GIVING BENEFIT OF THE DOUBT VERY DIFFICULT TO KNOW IF THESE SINGLE CENTRE PATIENTS ARE ALSO IN THE MULTI CENTRE PAPERS. Overlap with Farrow 2008, different predictors no concerns |
| **Brown 2013** | USA | Jan 2000 to Aug 2007 | GIVING BENEFIT OF THE DOUBT VERY DIFFICULT TO KNOW IF THESE SINGLE CENTRE PATIENTS ARE ALSO IN THE MULTI CENTRE PAPERS |
| **Clapp 2013** | USA | Jan 2007 to Dec 2010 | SOME OVERLAP WITH **BRAHMBHATT 2014**, **CARTER 2014** & **SUBRMANIAN 2013**, BUT DOESN'T MATTER AS DIFFERENT VARIABLE IN DATASHEET |
| **Salameh 2002** | USA | Jan 2000 to Jun 2001 | VERY SMALL OVERLAP WITH **LIANG 2013**, **SUBRAMANIAN 2013**, **CARTER 2014, BRAHMBHATT** **2014** AND **BROWN 2013** |
| **Farrow 2008** | USA | Oct 2003 to Sep 2007 | Open overlap with Liang 2013 but different predictors so nil concerns |
| **El-Gazzaz 2013** | USA | Dec 1991 to Aug 2007 | Unlikely overlap with Rosen 2003 or Ballem 2008 |
| **Rosen 2003** | USA | Jan 1996 to Mar 2001 | OVERLAP WITH **BALLEM 2008** FOR LAP CASES |
| **Ballem 2008** | USA | Jan 1996 to Dec 2001 | OVERLAP WITH **ROSEN 2003** FOR LAP CASES |
| **Zeichen 2013** | USA | Jul 2000 to Sep 2011 |  |
| **Fox 2013** | USA | Oct 2009 to Nov 2011 | Very different years, different authors unlikely overlap with Kanaan 2011 |
| **Kanaan 2011** | USA | 1995 and 2010 | Very different years, different authors unlikely overlap with Fox 2013 |
| **Snyder 2011** | USA | 1997 and 2002 | Overlap with Altom 2012 but different predictors so nil concern |
| **Gleysteen 2009** | USA | Feb 1988 to Sep 2001 | I don't think overlap with Altom - different centres involved |
| **Altom 2012** | USA | 1998 to 2002 | Overlap with Synder 2011, but different predictors so nil concernI don't think overlap with Gleysteen - different centres involved |
| **Singhal 2012** | USA | Jan 2001 to Feb 2010 |  |
| **Yannam 2011** | USA | Apr 2005 to Mar 2009 |  |
| **Kurian 2010** | USA | Apr 2001 to Apr 2009 |  |
| **Tsereteli 2008** | USA | 1993 to 2006 |  |
| **Lee 2008** | USA | 2000 to 2006 |  |
| **Saber 2008** | USA | Jul to Jul 2006 |  |
| **Raftopoulos 2003** | USA | 1994 and 2000 |  |
| **Gonzalez 2003** | USA | Nov 1995 to Oct 2000 |  |
| **Wright 2002a** | USA | Jan 1998 to Apr 2000 | OVERLAP WITH **WRIGHT 2002b** |
| **Wright 2002b** | USA | Jan 1998 to Apr 2000 | OVERLAP WITH **WRIGHT 2002a** |
| **Booth 2013** | USA | Feb 2000 to Oct 2011 | OVERLAP WITH **GARVEY 2012, GARVEY 2014, GARVEY 2016, GHALI 2012, CLEMENS 2013**, **GIORDANO 2016,** **GIORDANO 2017a**, **GIORDANO 2017b, GIORDANO 2017c** |
| **Garvey 2012** | USA | June 2002 to Nov 2010 | OVERLAP WITH **GARVEY 2014**, **GARVEY 2016,** **BOOTH 2013, GHALI 2012, CLEMENS 2013, GIORDANO 2016, GIORDANO 2017a, GIORDANO 2017b, GIORDANO 2017c** |
| **Giordano 2017a** | USA | Mar 2005 to Oct 2015 | OVERLAP WITH **GARVEY 2012, GARVEY 2014, GARVEY 2016, BOOTH 2013, GHALI 2012, CLEMENS 2013, GIORDANO 2016, GIORDANO 2017b**, **GIORDANO 2017c** |
| **Giordano 2017b** | USA | Mar 2005 to Oct 2015 | OVERLAP WITH **GARVEY 2012, GARVEY 2014, GARVEY 2016, BOOTH 2013, CLEMENS 2013, GHALI 2012**, **GIORDANO 2016, GIORDANO 2017a, GIORDANO 2017c** |
| **Giordano 2017c** | USA | Mar 2005 to Oct 2015 | OVERLAP WITH **GARVEY 2012**, **GARVEY 2014**, **GARVEY 2016,** **BOOTH 2013**, **CLEMENS 2013**, **GHALI 2012**, **GIORDANO 2016**, **GIORDANO 2017a**, **GIORDANO 2017b** |
| **Ghali 2012** | USA | Mar 2005 to Oct 2010 | OVERLAP WITH **GARVEY 2012, GARVEY 2014, GARVEY 2016, BOOTH 2013, CLEMENS 2013, GIORDANO 2016, GIORDANO 2017a, GIORDANO 2017b**, **GIORDANO 2017c** |
| **Clemens 2013** | USA | Jan 2008 to Mar 2011 | OVERLAP WITH **GARVEY 2012, GARVEY 2014, GARVEY 2016, BOOTH 2013, GHALI 2012**, **GIORDANO 2016, GIORDANO 2017a, GIORDANO 2017b**, **GIORDANO 2017c** |
| **Garvey 2014** | USA | Mar 2005 to Mar 2013 | OVERLAP WITH **GARVEY 2012, GARVEY 2016, CLEMENS 2013, BOOTH 2013, GHALI 2012**, **GIORDANO 2016, GIORDANO 2017a, GIORDANO 2017b**, **GIORDANO 2017c** |
| **Garvey 2016** | USA | Mar 2005 and Oct 2015 | OVERLAP WITH **GARVEY 2012, GARVEY 2014, CLEMENS 2013, BOOTH 2013, GHALI 2012**, **GIORDANO 2016, GIORDANO 2017a, GIORDANO 2017b**, **GIORDANO 2017c** |
| **Giordano 2016** | USA | Mar 2005 and Oct 2015 | OVERLAP WITH **GARVEY 2012, GARVEY 2014, GARVEY 2016, CLEMENS 2013, BOOTH 2013, GHALI 2012**, **GIORDANO 2017a, GIORDANO 2017b**, **GIORDANO 2017c** |
| **Ko 2009a** | USA | Sep 2004 to Sep 2007 | Some possible overlap with Ko 2009b but different predictors tested not an issue |
| **Ko 2009b** | USA | Aug 1996 to Jul 2007 | OVERLAP WITH **REID 2004**, Some possible overlap with Ko 2009a but different predictors tested not an issue |
| **Ujiki 2004** | USA | Apr 2000 to Feb 2003 | No overlap with Ko 2009a+b due to this being laparascopic only (Ko not laparoscopic) |
| **Reid 2004** | USA | 1997 to 2003 | OVERLAP WITH **KO 2009b** - Small group of patients - Likely included in Ko 2009 b |
| **Colon 2011** | USA | 2005 to 2009 | OVERLAP WITH **DANZIG 2016** (NOT KITAMURA 2013 AS INCISIONAL ONLY) |
| **Kitamura 2013** | USA | 2003 and 2009 | OVERLAP WITH **DANZIG 2016** (NOT COLON 2011 AS INCISIONAL ONLY) |
| **Groene 2016b** | USA | no dates | THERE IS OVERLAP HERE BUT THIS IS ONLY A SUBSET OF 44 PATIENTS OF THE IHMR ? OVERLAP WITH **WORMER 2013, GROENE 2016a** |
| **Colavita 2012** | USA | Sep 2007 to Jul 2011 | OVERLAP WITH **WORMER 2013**, **GROENE 2016a** |
| **Wormer 2013** | USA | Oct 2007 to Jun 2012 | OVERLAP WITH **COLAVITA 2012**, **GROENE 2016a** |
| **Groene 2016a** | USA | no dates | OVERLAP WITH **WORMER 2013, COLAVITA 2012** |
| **Johnson 2016** | USA | 2009 to 2013 |  |
| **Karipineni 2016** | USA | Aug 2010 to Jul 2013 |  |
| **Parent 2016** | USA | Jan 2010 to Jan 2016 | Possible small overlap with Sandvall 2016 but minimal |
| **Sandvall 2016** | USA | Nov 2006 to Nov 2010 | Possible small overlap with Parent 2016 but minimal |
| **Wennergren 2016** | USA | 2010 to 2012 | Possible overlap with some of the involved centres but only small proportion of patients |
| **Desai 2016** | USA | 2002 to 2014 | OVERLAP WITH **GHAZI 2011**, JUST LONGER PERIOD OF PATIENTS |
| **Ghazi 2011** | USA | 2002 and 2009 | OVERLAP WITH **DESAI 2016**, JUST SHORTER PERIOD OF PATIENTS |
| **Ecker 2016** | USA | 2007 and 2011 |  |
| **Danzig 2016** | USA | Jan 2002 to Dec 2010 | OVERLAP WITH **KITAMURA 2013, COLON 2011** |
| **Henry 2013** | USA | Jul 2008 to Oct 2011 | POSSIBLE OVERLAP WITH **WON 2015 AND RINALDI 2016,** BUT NOT AN ISSUE DIFFERENT PREDICTORS |
| **Rinaldi 2016** | USA | Jul 2011 to Mar 2013 | POSSIBLE OVERLAP WITH **WON 2015 AND HENRY 2013,** BUT NOT AN ISSUE DIFFERENT PREDICTORS |
| **Won 2015** | USA | May 2011 to Nov 2013 | POSSIBLE OVERLAP WITH **RINALDI 2016 AND HENRY 2013**, BUT NOT AN ISSUE DIFFERENT PREDICTORS |
| **Hultman 2014** | USA | 2000 to 2010 |  |
| **Satterwhite 2012** | USA | Sep 2002 to Feb 2010 |  |
| **Iqbal 2007** | USA | Oct 1991 to Oct 2003 |  |
| **Krpata 2013** | USA | Sep 2005 and Jan 2012 | I don't thin overlap these are ECF repairs other case papers are not |
| **Itani 2012** | USA | no dates | OVERLAP WITH **ROSEN 2012** (ROSEN WAS AN INTERIM ANALSIS WITH LESS PATIENTS AND LESS FOLLOW UP TIME) |
| **Anthony 2000** | USA | Oct 1991 to Sep 1995 |  |
| **Abdelfatah 2015** | USA | Oct 2004 to Jun 2008 |  |
| **Roth 2015** | USA | 2007 and 2010 | Multicentre unlikely ovelap with Johnson 2016 |
| **Rosen 2017** | USA | Feb 2011 to Dec 2014 |  |
| **Chand 2014** | USA | Aug 2010 to Oct 2011 |  |
| **Sailes 2010** | USA | Oct 1996 to Oct 2006 |  |
| **DiCocco 2009** | USA | Oct 1993 to Dec 2008 |  |
| **Clarke 2010** | USA | no dates |  |
| **Lin 2009** | USA | May 2005 to Feb 2008 |  |
| **Candage 2008** | USA | May 2004 to Oct 2007 |  |
| **Heartsill 2005** | USA | 1996 and 2000 | No overlap with Franklin 2004 as Heartsill has open surgey |
| **Perrone 2005** | USA | May 2000 to Dec 2003 |  |
| **Franklin 2004** | USA | Feb 1991 to Nov 2002 | No overlap with Heart 2005 as Franklin has Lap patients |
| **Clark 2001** | USA | Oct 2003 to Dec 1996 |  |
| **Davidson 2009** | USA | 1999 and 2005 |  |
| **Gassman 2015** | USA | May 2008 to Jul 2011 |  |
| **Holihan 2015** | USA | 2000 to 2012 | OVERLAP WITH **HOLIHAN 2016** (MODEL DEV AND INTERNAL VALIDATION), SOME OVERLAP WITH **BONDRE 2016** **BUT** SOME DIFFERENT CENTRES AND DIFFERENT DATES OF RECRUITMENT SO DISCOUNT |
| **Bondre 2016** | USA | Jan 2010 to Dec 2011 | OVERLAP WITH **HOLIHAN 2016** (EXTERNAL VALIDATION), SOME OVERLAP WITH **HOLIHAN 2015** **BUT** SOME DIFFERENT CENTRES AND DIFFERENT DATES OF RECRUITMENT SO DISCOUNT |
| **Holihan 2016** | USA | 2009-2010 | **OVERLAP TRICKY!!!** MODEL DEVELOPMENT AND INTERNAL VALIDATION OVERLAP WITH **HOLIHAN 2015,** EXTERNAL VALIDATION OVERLAP WITH **BONDRE 2016** |
| **Diamond 2015** | USA | 2006 to 2011 |  |
| **Flum 2003** | USA | 1987 to 1999 |  |
| **Baucom 2016** | USA | no dates |  |
| **Bender 2016** | USA | Jan 1995 to Jun 2014 |  |
| **Heimann 2017** | USA | Jan 1976 to Dec 2014 |  |
| **Shankar 2017** | USA | Jan 1998 to Dec 2008 |  |
| **Yao 2016** | USA | Aug 2005 to Jul 2014 |  |
| **Hadeed 2011** | Germany | Jan 2005 to Sep 2009 |  |
