# Supplementary material for: Identifying predictors of ventral hernia recurrence: systematic review and meta-analysis
Source: BJS Open. 2021 Apr 11;5(2):zraa071. doi: 10.1093/bjsopen/zraa071 (PMC8038271; doi:10.1093/bjsopen/zraa071)
Supplement: zraa071_Supplementary_Data [file zraa071_supplementary_data.zip › OnlineResource4.Definitions.docx]

Online Resource 4 – Definitions of Recurrence

| **Definitions** |
| --- |
| Yes - palpable lump at the site of previous repair |
| Yes - Abdo wall defect detectable on examination or imaging |
| Yes - Hernias at the same location |
| Yes - defined by clinical examination |
| Yes - protruding bulge whilst doing a valsalva at previous repair site |
| Yes - ‘‘Recurrence during follow-up’’ of an incisional hernia was defined as a repeat incisional hernia operation, abdominal wall weakness in the area of the incision, or localized bulging upon coughing. |
| Yes - defect of the midline aponeurosis around the umbilicus at the site where the operation had been performed |
| Yes - protrusion of contents of the abdominal cavity through a defect in the abdominal wall at the site of repair |
| Yes - bulge at hernia repair site |
| Yes - palpable lump at the site of previous repair |
| Yes - bulge at hernia repair site |
| Yes - new hernia within 7cm of the repair |
| Yes - central tissue eventration when hernia sac extends beyond the boundaries of the anterior abdom wall |
| Yes - abnormal contour associated with a fascial defect |
| Yes - recurrence involved more than one side of the hernia or large than 2.5cm |
| Yes - bulge at hernia repair site |
| Yes - hernia at repair site |
| Yes - defect in the midline, parastomal area, at the flap harvest site. |
| Yes - bulge/reoperation |
| Yes - recurrent fascial defect on examination or scan |
| Yes - palpable defect |
| Yes - patient complained of simliar symptom to what they had before the repair |
| Yes - isolated palpable defect at the site of the previous repair |
| Yes - recurrent bulge in the supine and standing positions |
| Yes - any gap in the abdominal wall identified on imaging |
| Yes - Reoperation |
| Yes - reoperation at the site of previous hernia repair |
| Yes - protrusion of fat through a defect in the abdominal wall at the site of previous repair of an abdominal wall hernia. |
| Yes - contour abnormality with a fascial defect |
| Yes - hernia at incision site OR ileostomy closure site |
| Yes - but defined as whether found during f/u |
| Yes - reoperation at the site of previous hernia repair |
| Yes - re-operation rate |
| Yes - fascial defect with contour abnormality |
| Yes - Radiological evidence of recurrence |
| Yes - palpable bulge |
| Yes - fascial defect with contour abnormality |
| Yes - bulge or defect at the site of VH repair |
| Yes - fascial defect with contour abnormality |
| Yes - re-operation rate |
| Yes - fascial defect with protrusion of bowel |
| Yes - fascial edges of defect palpable |
| Yes - re-operation, examination, CT, telephone |
| Yes - abdo wall defect |
| Yes - reoperation or clinical/radiological evidence of recurrence |
| Yes - fascial defect |
| Yes - palpable lump at the site of previous repair |
| Yes - palpable lump within 7cm of hernia repair site |
| Yes - requiring another op OR a significant bulge |
| Yes - defect in abdo wall at site of previous hernia |
| Yes - bulge at site of op getting bigger with coughing |
| Yes - defect in abdo wall at site of previous hernia |
| Yes - defect in abdo wall at site of previous hernia |
| Yes - abdo wall defect |
| Yes - central or port site |
| Yes - tissue protruding beyond the anterior plane of the anterior rectus fascia on CT scan |
| Yes - a palpable defect on exam as noted by the attending surgeon, further confirmed by CT imaging in all cases. |
| Yes - the presence of a bulge on physical examination, imaging, or by patient self-reporting |
| Yes - any abdominal wall gap with or without bulge that is not covered by mesh in the area of a postoperative scar |
| Yes - a symptomatic herniation or a herniation was detected via abdominal ultrasonography |
| Yes - a true hernia recurrence as herniation of bowel or omentum through a defect in the biologic mesh or through a defect at the mesh/fascial interface after the initial operation. |
| Yes - a clinically detectable defect, associated with the protrusion of viscera on straining. |
| Yes - any fascial defect that was palpable or detected by ultrasound examination and was located within 7 cm of the site of hernia repair |
| Yes - as the presence of a defect on the central part of the midline aponeurosis around the umbilicus, where the operation had been performed previously. |
| Yes - the presence of a defect on the central part of the midline aponeurosis where the operation had been performed previously |
| Yes - a defect of the midline aponeurosis around the umbilicus at the site where the operation had been performed |
| **66 studies gave a definition for recurrence** |
|  |
| **Definition 1** |
| Yes - palpable lump at the site of previous repair |
| Yes - palpable lump at the site of previous repair |
|  |
| **Definition 2** |
| Yes - Abdo wall defect detectable on examination or imaging |
|  |
| **Definition 3** |
| Yes - Hernias at the same location |
| Yes - hernia at repair site |
|  |
| **Definition 4** |
| Yes - defined by clinical examination |
|  |
| **Definition 5** |
| Yes - protruding bulge whilst doing a valsalva at previous repair site |
|  |
| **Definition 6** |
| Yes - defect of the midline aponeurosis around the umbilicus at the site where the operation had been performed |
| Yes - as the presence of a defect on the central part of the midline aponeurosis around the umbilicus, where the operation had been performed previously. |
| Yes - the presence of a defect on the central part of the midline aponeurosis where the operation had been performed previously |
| Yes - a defect of the midline aponeurosis around the umbilicus at the site where the operation had been performed |
|  |
| **Definition 7** |
| Yes - protrusion of contents of the abdominal cavity through a defect in the abdominal wall at the site of repair |
| Yes - protrusion of fat through a defect in the abdominal wall at the site of previous repair of an abdominal wall hernia. |
|  |
| **Definition 8** |
| Yes - bulge at hernia repair site |
| Yes - bulge at hernia repair site |
|  |
| **Definition 9** |
| Yes - new hernia within 7cm of the repair |
| Yes - palpable lump within 7cm of hernia repair site |
|  |
| **Definition 10** |
| Yes - central tissue eventration when hernia sac extends beyond the boundaries of the anterior abdom wall |
|  |
| **Definition 11** |
| Yes - abnormal contour associated with a fascial defect |
| Yes - contour abnormality with a fascial defect |
| Yes - fascial defect with contour abnormality |
| Yes - fascial defect with contour abnormality |
| Yes - fascial defect with contour abnormality |
|  |
| **Definition 12** |
| Yes - recurrence involved more than one side of the hernia or large than 2.5cm |
|  |
| **Definition 13** |
| Yes - defect in the midline, parastomal area, at the flap harvest site. |
|  |
| **Definition 14** |
| Yes - bulge/reoperation |
| Yes - requiring another op OR a significant bulge |
|  |
| **Definition 15** |
| Yes - palpable defect |
| Yes - fascial edges of defect palpable |
|  |
| **Definition 16** |
| Yes - patient complained of simliar symptom to what they had before the repair |
|  |
| **Definition 17** |
| Yes - isolated palpable defect at the site of the previous repair |
|  |
| **Definition 18** |
| Yes - recurrent bulge in the supine and standing positions |
|  |
| **Definition 19** |
| Yes - any gap in the abdominal wall identified on imaging |
| Yes - Radiological evidence of recurrence |
|  |
| **Definition 20** |
| Yes - Reoperation |
| Yes - re-operation rate |
| Yes - re-operation rate |
|  |
| **Definition 21** |
| Yes - reoperation at the site of previous hernia repair |
| Yes - reoperation at the site of previous hernia repair |
|  |
| **Definition 22** |
| Yes - hernia at incision site OR ileostomy closure site |
|  |
| **Definition 23** |
| Yes - ‘‘Recurrence during follow-up’’ of an incisional hernia was defined as a repeat incisional hernia operation, abdominal wall weakness in the area of the incision, or localized bulging upon coughing. |
|  |
| **Definition 24** |
| Yes - but defined as whether found during f/u |
|  |
| **Definition 25** |
| Yes - palpable bulge |
|  |
| **Definition 26** |
| Yes - bulge or defect at the site of VH repair |
|  |
| **Definition 27** |
| Yes - fascial defect with protrusion of bowel |
|  |
| **Definition 28** |
| Yes - re-operation, examination, CT, telephone |
|  |
| **Definition 29** |
| Yes - abdo wall defect |
| Yes - fascial defect |
| Yes - abdo wall defect |
|  |
| **Definition 30** |
| Yes - reoperation or clinical/radiological evidence of recurrence |
|  |
| **Definition 31** |
| Yes - defect in abdo wall at site of previous hernia |
| Yes - defect in abdo wall at site of previous hernia |
| Yes - defect in abdo wall at site of previous hernia |
|  |
| **Definition 32** |
| Yes - bulge at site of op getting bigger with coughing |
|  |
| **Definition 33** |
| Yes - central or port site |
|  |
| **Definition 34** |
| Yes - tissue protruding beyond the anterior plane of the anterior rectus fascia on CT scan |
|  |
| **Definition 35** |
| Yes - a palpable defect on exam as noted by the attending surgeon, further confirmed by CT imaging in all cases. |
|  |
| **Definition 36** |
| Yes - the presence of a bulge on physical examination, imaging, or by patient self-reporting |
|  |
| **Definition 37** |
| Yes - any abdominal wall gap with or without bulge that is not covered by mesh in the area of a postoperative scar |
|  |
| **Definition 38** |
| Yes - a symptomatic herniation or a herniation was detected via abdominal ultrasonography |
|  |
| **Definition 39** |
| Yes - a true hernia recurrence as herniation of bowel or omentum through a defect in the biologic mesh or through a defect at the mesh/fascial interface after the initial operation. |
|  |
| **Definition 40** |
| Yes - a clinically detectable defect, associated with the protrusion of viscera on straining. |
|  |
| **Definition 41** |
| Yes - any fascial defect that was palpable or detected by ultrasound examination and was located within 7 cm of the site of hernia repair |
