# Supplementary material for: Identifying predictors of ventral hernia recurrence: systematic review and meta-analysis
Source: BJS Open. 2021 Apr 11;5(2):zraa071. doi: 10.1093/bjsopen/zraa071 (PMC8038271; doi:10.1093/bjsopen/zraa071)
Supplement: zraa071_Supplementary_Data [file zraa071_supplementary_data.zip › OnlineResource5.Detection.docx]

Online Resource 5 – Methods of Detecting Recurrence

| **Method of detecting recurrence** | **Grouping** |
| --- | --- |
| adhoc Clinical assessment | **MI** |
| Clinical assesment +/- CT +/- Telephone call | MI |
| Clinical Assessment | **CA** |
| Clinical assessment + CT | CC |
| Clinical assessment + medical records | **MI** |
| Clinical assessment + telephone call | CT |
| Clinical assessment + USS | CU |
| Clinical assessment +/- CT | CC |
| Clinical assessment +/- CT +/- medical records | MI |
| Clinical Assessment +/- CT +/- re-operation | MI |
| Clinical assessment +/- CT +/- telephone call | MI |
| Clinical assessment +/- CT/USS | CI |
| Clinical assessment +/- CT/USS +/- re-operation | MI |
| Clinical Assessment +/- CT/USS +/- telephone call | MI |
| Clinical Assessment +/- medical records | **MI** |
| Clinical assessment +/- medical records +/- telephone call +/- CT | MI |
| Clinical Assessment +/- Questionnaire | CQ |
| Clinical Assessment +/- Questionnaire +/- Telephone call | MI |
| Clinical assessment +/- re-operation | **MI** |
| Clinical Assessment +/- telephone call | CT |
| Clinical assessment +/- telephone call +/- questionnaire | MI |
| Clinical Assessment +/- telephone call +/- questionnaire +/- clinical notes | MI |
| Clinical assessment +/- US | CU |
| Clinical assessment +/- US/CT +/- re-operation | MI |
| Clinical Assessment +/- USS/CT | CI |
| Clinical Assessment +/- USS/CT +/- re-operation | MI |
| Clinical assessment/medical records/imaging | **MI** |
| Clinical assessment/telephone | **CT** |
| Clinical assessment/telephone&Questionnaire | **MI** |
| Clinical assessment+/-CT/re-operation | MI |
| Clinical notes & records + telephone call +/- clinical assessment +/- CT | MI |
| Clinical records +/- telephone call | CT |
| Medical records | MR |
| Medical records +/- Clinical assessment | **MI** |
| Medical records +/- clinical Assessment +/- CT | **MI** |
| Medical records +/- questionnaire +/- GP records +/- clinical assessment | MI |
| Medical records +/- Telephone +/- Clinical Assessment | **MI** |
| Medical records +/- telephone call | **MI** |
| Medical records +/- telephone call +/- CT +/- reoperation | MI |
| No information | **NI** |
| Prospectively maintained database | **MI** |
| Prospectively maintained database +/- Medical records | **MI** |
| Questionnaire | **MI** |
| Questionnaire +/- Clinical Assessment + US | MI |
| Questionnaire +/- Clinical Assessment +/- CT/USS | MI |
| Questionnaire +/- telephone call +/- clinical Assessment | MI |
| Questionnaire +/- telephone call +/- clinical assessment +/- CT | MI |
| Questionnaire +/- telephone call +/- clinical examination +/- CT/USS | MI |
| Questionnaire/GP records | **MI** |
| Re-operation | RO |
| Re-operation rate | RO |
| Re-operation rate +/- Clinical assessment +/- CT/USS | MI |
| Telephone +/- Clinical assessment | CT |
| Telephone +/- clinical assessment +/- CT | MI |
| Telephone call | **MI** |
| Telephone call + Questionnaire | MI |
| Telephone call +/- Clinical assessment | CT |
| Telephone call +/- Clinical assessment +/- CT | MI |
| Telephone call +/- Clinical assessment +/- CT/USS | MI |
| Telephone call +/- clinical assessment +/- medical records | MI |
| USS +/- CT | **IU/C** |
|  |  |
|  |  |
| **Group codes** |  |
|  |  |
| Imaging only with CT - IC (not many studies) |  |
| Imaging only with US - IU (not many studies) |  |
| **Imaging only with USS or CT - IU/C** |  |
| **Clinical Assessment - CA** |  |
| Clinical assessment combined with CT - CC |  |
| Clinical assessment combined with USS - CU |  |
| Clinical assessment combined with CT or USS - CI |  |
| Clinical assessment combined with telephone - CT |  |
| Clinical assessment combined with Questionnaire - CQ |  |
| Medical records synonymous with clinical records & clinical notes - MR |  |
| Re-operation - RO |  |
| Mixture - MI |  |
|  |  |
| The Mixture group with include a lot - eg. Clinical assessment + clinical notes + telephone, OR Telephone + Questionnaire |  |
|  |  |
| **NI - No Information** |  |
