# Supplementary material for: Identifying predictors of ventral hernia recurrence: systematic review and meta-analysis
Source: BJS Open. 2021 Apr 11;5(2):zraa071. doi: 10.1093/bjsopen/zraa071 (PMC8038271; doi:10.1093/bjsopen/zraa071)
Supplement: zraa071_Supplementary_Data [file zraa071_supplementary_data.zip › OnlineResource6.Overall.docx]

## Online Resource 6

## Patient demographics

## Sex

## Age

## BMI

# Co-morbidities

## Smoker

## Diabetes

##

## Hypertension

##

## Cardiac

## BPH

## COPD

## ASA 3-4 Vs ASA 1-2

## Immunosuppression

**3.0 Hernia related**

**3.1 Contaminated**


**3.2 VHWG**

**3.3 Previous wound infection**

**3.4 Hernia Area**

**3.5 Hernia width**

**3.6 Midline Vs Lateral**

**3.7 Incisional Vs Primary**

- 1. **Recurrent Vs Primary**

1. **Intra-operative**

**4.1 Human biologic Vs Porcine biologic**

**4.2 Biologic Vs Synthetic**

**4.3 Bridging Vs Primary closure**

**4.4 Component separation Vs no Component Separtion**

**4.5 Laparoscopic Vs Open Surgery**

**4.6 Lightweight mesh Vs other mesh**

**4.7 Mesh Vs Suture repair**

**4.8 Position of mesh**

1. **Post-operative**
   1. **Post-operative complication**

- 1. **Surgical site Occurrence**

- 1. **Wound infection**

- 1. **Seroma**

- 1. **Wound dehiscence**
