# Supplementary material for: Identifying predictors of ventral hernia recurrence: systematic review and meta-analysis
Source: BJS Open. 2021 Apr 11;5(2):zraa071. doi: 10.1093/bjsopen/zraa071 (PMC8038271; doi:10.1093/bjsopen/zraa071)
Supplement: zraa071_Supplementary_Data [file zraa071_supplementary_data.zip › OnlineResource7.docx]

**Online Resource 7**

# AAA repair – only 3 studies (hernia related variable)

# Concurrent GI – not enough studies (Intra-operative variable)

# Panniculectomy - not enough studies (Intra-operative variable)

# Tack Vs Suture - not enough studies (Intra-operative variable)
