# Supplementary material for: Identifying predictors of ventral hernia recurrence: systematic review and meta-analysis
Source: BJS Open. 2021 Apr 11;5(2):zraa071. doi: 10.1093/bjsopen/zraa071 (PMC8038271; doi:10.1093/bjsopen/zraa071)
Supplement: zraa071_Supplementary_Data [file zraa071_supplementary_data.zip › OnlineResource8.DeletedPredictors.docx]

Online Resource 8 – Deleted Predictors

| **PaperID** | **Area** | **Column1** | **PrognosticFactor** |
| --- | --- | --- | --- |
| Christoffersen 2013 | Absorable Vs Non-Abs Suture | OUT | Slow-absorbable Vs Fast absorbable suture] |
| Fayezizadeh 2016 | Admission | OUT | ICU admission Vs no ICU admission |
| Hauters 2017 | Hernia Area | Correct - BUT OUT - only data from the paper | Surface area of defect |
| Notash 2007 | Hernia Area | Correct - OUT | Defect area+1cm^2 Vs defect area |
| Oma 2017 | Hernia Area | Correct - Area+10cm^2/Area - OUT | Hernia defect area+10cm^2 Vs hernia defect area |
| Bontinck 2014 | Hernia width | Correct - width+1/width - OUT | Hernia defect width+1cm Vs Hernia defect width |
| Helgstrand 2013 | Hernia width | Correct - ? Subgroup (Probably OUT) | Hernia defect width 7-15cm Vs 0-2cm |
| Helgstrand 2013 | Hernia width | Correct - ? Subgroup (Probably OUT) | Hernia defect width >20cm Vs 0-2cm |
| Helgstrand 2013 | Hernia width | Correct - ? Subgroup (Probably OUT) | Hernia defect width 15-20cm Vs 0-2cm |
| Kokotovic 2016 | Hernia width | Correct - ? Subgroup (Probably OUT) | Hernia defect width 7-15cm Vs 0-2cm |
| Petro 2016 | Hernia width | Correct - width+10/width - OUT | Hernia defect width +10cm Vs hernia defect width |
| Ferrarese 2016 | Location | Correct - OUT nil other | Suprapubic Vs Epigastric |
| Ferrarese 2016 | Location | Correct - OUT nil other | Subcostal Vs Suprapubic |
| Ferrarese 2016 | Location | Correct - OUT nil other | Epigastric Vs Lumbar |
| Ferrarese 2016 | Location | Correct - OUT nil other | Epigastric Vs Non Epigastric |
| Ferrarese 2016 | Location | Correct - OUT nil other | Suprapubic Vs Non suprapubic |
| Ferrarese 2016 | Location | Correct - OUT nil other | Suprapubic Vs Lumbar |
| Ferrarese 2016 | Location | Correct - OUT nil other | Subcostal Vs Epigastric |
| Ferrarese 2016 | Location | Correct - OUT nil other | Lumbar Vs Non lumbar |
| Ferrarese 2016 | Location | Correct - OUT nil other | Subcostal Vs Non subcostal |
| Ferrarese 2016 | Location | Correct - Subcostal Vs Lumbar - OUT | Subcostal Vs Lumbar |
| Keating 2016 | Location | Correct - Epigastric Vs Umbilical - OUT | Umbilical Vs Epigastric |
| Moreno-Egea 2012a | Location | Correct - OUT nil other | Iliac IH Vs Lumbar IH |
| Oma 2017 | Location | Correct - OUT only one other | Incisional Vs Epigastric |
| Basoglu 2004 | Material | Correct - PP Vs PE - OUT only 2 | Polypropylene Vs Polyester |
| Brown 2013 | Material | Correct - ePTFE vs non-ePTFE - OUT - only one | ePTFE Vs non-ePTFE |
| Carter 2014 | Material | Correct - PTFE Vs PEL - OUT only 2 | Polytetraflouroethylene Vs Polyethylene |
| Carter 2014 | Material | Correct - PP Vs PEL - OUT only 2 | Polypropylene Vs polyethylene |
| Carter 2014 | Material | Correct - PP Vs PTFE - OUT only 2 | Polytetraflouroethylene Vs Polypropylene |
| Hauters 2017 | MD Ratio | Correct - OUT no other papers have this variable | MD-Ratio 13-16 Vs MD-Ratio 9-12 |
| Hauters 2017 | MD Ratio | Correct - OUT no other papers have this variable | MD-Ratio 9-12 Vs MD-Ratio ≦8 |
| Hauters 2017 | MD Ratio | Correct - OUT no other papers have this variable | MD-Ratio ≥17 Vs MD-Ratio ≦8 |
| Hauters 2017 | MD Ratio | Correct - OUT no other papers have this variable | MD-Ratio ≥17 Vs MD-Ratio 9-12 |
| Hauters 2017 | MD Ratio | Correct - OUT no other papers have this variable | MD-Ratio 13-16 Vs MD-Ratio ≦8 |
| Hauters 2017 | MD Ratio | Correct - OUT no other papers have this variable | MD-Ratio ≥17 Vs MD-Ratio 13-16 |
| Hauters 2017 | MD Ratio | Correct - OUT no other papers have this variable | M/D ratio |
| Groene 2016a | Medium Vs Heavy | Correct - OUT no other papers have this variable | Mediumweight Vs Heavyweight |
| Hauters 2017 | Mesh overlap | Correct - OUT >/<5cm overlap - only 1 paper | Mesh overlap 5cm Vs 4cm |
| Hauters 2017 | Mesh overlap | Correct - OUT ?only 1 so OUT | Mesh overlap |
| Hauters 2017 | Mesh overlap | Correct - OUT >/<5cm overlap - only 1 paper | Mesh overlap 5cm Vs 3cm |
| Hauters 2017 | Mesh overlap | Correct - OUT >/<5cm overlap - only 1 paper | Mesh overlap ≥6cm Vs 4cm |
| Hauters 2017 | Mesh overlap | Correct - OUT >/<3cm - only 2 - OUT | Mesh overlap 4cm Vs 3cm |
| Hauters 2017 | Mesh overlap | Correct - OUT >/<5cm overlap - only 1 paper | Mesh overlap ≥6cm Vs 3cm |
| Hauters 2017 | Mesh overlap | Correct - OUT >/<5cm overlap - only 1 paper | Mesh overlap ≥6cm Vs 5cm |
| Lambrecht 2014 | Mesh overlap | Correct - ?only 1 so OUT | Overlap coefficient |
| Tsimoyiannis 2008 | Mesh overlap | Correct - >/<3cm - only 2 - OUT | Mesh overlap 2.5cm Vs 4.5cm |
| Bensaadi 2014 | Mesh related | Correct - BUT OUT AS ONLY 1 | Ventralex vs Biomesh Cabs'Air |
| Keating 2016 | Mesh related | Correct - BUT OUT AS ONLY 1 | Medium V-Patch Vs Small V-Patch |
| Keating 2016 | Mesh related | Correct - BUT OUT AS ONLY 1 | Large V-Patch Vs Small V-Patch |
| Keating 2016 | Mesh related | Correct - BUT OUT AS ONLY 1 | Large V-Patch Vs Medium V-Patch |
| Martinez 2017 | Mesh related | Correct - BUT OUT AS ONLY 1 | Kugel patch Vs Ventrio patch |
| Martinez 2017 | Mesh related | Correct - BUT OUT AS ONLY 1 | Kugel patch Vs Ventrio patch |
| Martinez 2017 | Mesh related | Correct - BUT OUT AS ONLY 1 | Kugel patch Vs Ventrio patch |
| Pawlak 2016 | Mesh related | Correct - BUT OUT AS ONLY 1 | Physiomesh (rigid) vs Ventralight(elastic) |
| Tandon 2016 | Mesh related | Correct - BUT OUT AS ONLY 1 | Parietex Vs Dynamesh |
| Cox 2016 | No. Comorbidities | Correct - BUT OUT AS ONLY 1 | ≥2 Co-morbidity Vs <2 co-morbidities |
| Bencini 2009 | Operation time | Correct - BUT OUT AS ONLY 1 | Operating time (Continuous variable, Multivariable HR analysis) |
| Fischer 2014 | Operation time | Corrct - BUT OUT AS ONLY 1 | Prolonged Operating time Vs not prolonged |
| Greenstein 2008 | Operation time | Correct - >/<120min - OUT as only 2 | Operative time (>/< 120 mins) |
| Hornby 2015 | Operation time | Correct - >/<90min - OUT as only 1 | Duration of operation >90mins Vs <90mins |
| Rosen 2013 | Operation time | Correct - >/<90min - OUT as only 1 | Operating time+60mins Vs Operating time |
| Slater 2015c | Operation time | Correct - >/<120min - OUT as only 2 | Operating time+120mins Vs Operating time |
| Heimann 2017 | Other disease | Correct - BUT OUT AS ONLY 1 | Crohn Vs Ulcerative colitis |
| Heimann 2017 | Other disease | Correct - BUT OUT AS ONLY 1 | Hypoalbuminaemia Vs no hypoalbuminaemia |
| Heimann 2017 | Other disease | Correct - BUT OUT AS ONLY 1 | Anaemia Vs no Anaemia |
| Shankar 2017 | Other disease | Correct - Liver dis/no Liver dis - OUT ONLY 2 | Liver disease Vs no Liver disease |
| Vidovic 2006 | Other disease | Correct - BUT OUT AS ONLY 1 | No Chronic disease Vs Any Chronic disease |
| Caruso 2017 | Other Fixation | Correct - Non-absorbable Vs absorbable - OUT only 3 | Titanium staples Vs absorbatacks |
| Caruso 2017 | Other Fixation | Correct - BUT OUT AS ONLY 1 | Titanium staples Vs titanium coils |
| Dalenback 2013 | Other Fixation | Correct - BUT OUT AS ONLY 1 | Mayo Vs Single row |
| Dalenback 2013 | Other Fixation | Correct - BUT OUT AS ONLY 1 | Double row suture Vs Single row |
| Dalenback 2013 | Other Fixation | Correct - BUT OUT AS ONLY 1 | Mayo Vs double row suture |
| Hauters 2017 | Other Fixation | Correct - BUT OUT AS ONLY 1 | Transfascial sutures |
| Hauters 2017 | Other Fixation | Correct - BUT OUT AS ONLY 1 | No Transfascial sutures Vs Transfascial sutures |
| Hornby 2015 | Other Fixation | Correct - BUT OUT AS ONLY 1 | Protack Vs no protack |
| Lambrecht 2014 | Other Fixation | Incorrect - BUT OUT AS ONLY 1 | Ingrowth area |
| Muysoms 2013 | Other Fixation | Correct - BUT OUT AS ONLY 1 | Double-crown tacks vs Sutures and Tacks |
| Baucom 2016 | Pain | Correct - OUT ONLY 3 | Post-op Symptomatic pain Vs non symptomatic pain |
| Carter 2014 | Pain | Correct - OUT ONLY 3 | Post chronic pain Vs no chronic pain |
| Groene 2016b | Pain | Correct - OUT ONLY 2 | Pre-symptomatic pain Vs non symptomatic pain |
| Lauscher 2013 | Pain | Correct - OUT only 1 paper | Pre-chronic pain Vs no chronic pain |
| Helgstrand 2013 | Position of mesh | Correct - ? OUT as planes are combined | Onlay mesh Vs Sublay mesh (retrorectus or pre-peritoneal) |
| Helgstrand 2013 | Position of mesh | Correct - - ? OUT as planes are combined | Onlay mesh Vs Sublay mesh (retrorectus or pre-peritoneal) |
| Helgstrand 2013 | Position of mesh | Correct - - ? OUT as planes are combined | Sublay mesh (retrorectus or pre-peritoneal) Vs Intraperitoneal mesh |
| Petro 2015 | Position of mesh | OUT | Prior intra-abdominal mesh Vs no prior intra-abdominal mesh |
| Prasad 2011 | Position of mesh | OUT | TAPP Lap repair VS IPOM Lap repair |
| Rosen 2013 | Position of mesh | Out - Only 2 | Sandwich repair Vs Intra-peritoneal mesh |
| Slater 2015c | Position of mesh | OUT - difficult to tell what the sublay is reffered too | Sublay mesh Vs non-sublay mesh |
| Slater 2015c | Position of mesh | OUT- difficult to tell ? intraperitoneal mesh is referrenced too | Intra-peritoneal mesh Vs non-intra-peritoneal mesh |
| Slater 2015c | Position of mesh | OUT - difficult to tell what the onlay is reffered too | Onlay mesh Vs non-onlay mesh |
| Oma 2017 | Pregnancy | Correct - PROB OUT AS ONLY 3 | Pregnancy Vs No pregnancy |
| Booth 2013 | Previous abdomial surgeries | Correct - Prior abdo surg y/n OUT ONLY 3 | Previous abdominal surgery Vs no previous surgery |
| Diamond 2015 | Previous abdomial surgeries | Correct - ≥4/<4PriorSurg (PORB OUT ONLY 2) | ≥4prior abdominal operations Vs <4prior abdominal operations |
| Rosen 2013 | Previous abdomial surgeries | Correct - PriorSurg+1VsPriorSurg (OUT ONLY 1) | No previous abdominal surgeries+1 Vs no previous abdominal surgeries |
| Flum 2003 | Primary Vs Recurrent | Correct - ThirdIHrepairVsSecondIHrepair (4 - OUT) | 2nd IH recurrence Vs 1st IH recurrence repair |
| Flum 2003 | Primary Vs Recurrent | Correct - SecondIHrepairVsFirstIHrepair (6 - OUT) | 1st IH recurrence Vs primary IH repair |
| Flum 2003 | Primary Vs Recurrent | Correct - ThirdIHrepairVsFirstIHrepair - OUT ONLY 2 | 2nd IH recurrence VS primary IH repair |
| Gecim 1996 | Primary Vs Recurrent | Correct - >2priorrepairs Vs <=2 (OUT ONLY 2) | Previous hernia repair >2 Vs Previous repir <=2 |
| Gecim 1996 | Primary Vs Recurrent | Correct - >=2priorrepairs Vs <2 (OUT ONLY 2) | Previous hernia repair >1 Vs Previous repir <=1 |
| Holihan 2015 | Primary Vs Recurrent | Correct - FourthVHrepairVsThirdVHrepair (OUT ONLY 2) | 3rd IH repair Vs 2nd IH repair |
| Holihan 2015 | Primary Vs Recurrent | Correct - FourthVHrepairVsSecondVHrepair (OUT ONLY 2) | 3rd IH repair VS primary VH repair |
| Holihan 2015 | Primary Vs Recurrent | Correct - FourthVHrepairVsFirstVHrepair (OUT ONLY 1) | 3rd IH repair VS primary VH repair |
| Krpata 2013 | Primary Vs Recurrent | Correct - Repair+1 Vs Repair (PROB OUT ONLY 3) | No previous hernia repair +1 Vs No previous hernia repairs |
| Wink 2014 | Smoker | Correct - Only 2 prob OUT | Ex-smoker Vs non-smoker |
| Bencini 2009 | Surgeon experience | Correct - ONLY 1 Prob OUT | Surgeon experience <10 procedures Vs >10 procedures |
| Gecim 1996 | Surgeon experience | Correct - ONLY 2 Prob OUT | Registrar Vs Consultant |
| Kokotovic 2016 | Tack Vs NoTack | Correct - OUT only 1 paper | Tack fixation Vs non-tack fixation |
| Sharma 2011 | Tack Vs Suture | Incorrect - OUT only 1 | Tacks Vs Suture |
| Wassenaar 2010 | Tack Vs Suture | Incorrect - OUT only 1 | Tacks vs Suture fixation |
| Basta 2015 | VHWG | Incorrect - separate - prob OUT only 1 | Modified VH grade 3 Vs modified VH grade 2 |
| Slater 2015a | VHWG | Correct - VHWG+1/VHWG (Prob OUT, Only 4) | VHWG modified grade+1 Vs VHWG modified grade |
| Slater 2015a | VHWG | Correct - VHWG+1/VHWG (Prob OUT, Only 4) | VHWG grade+1 Vs VHWG grade |
| Slater 2015c | VHWG | Correct - VHWG+1/VHWG (Prob OUT, Only 4) | VHWG modified grade+1 Vs VHWG modified grade |
| Slater 2015c | VHWG | Correct - VHWG+1/VHWG (Prob OUT, Only 4) | VHWG grade+1 Vs VHWG grade |
| Booth 2013 | Violated rectus | Correct- OUT only 4 | Violated rectus Vs Non-violated rectus |
| Krpata 2012 | Zno Group | OUT | Anterior Comp Sep Vs Posterior Comp Sep |
| Wink 2014 | Zno Group | OUT | Bilateral C/S Vs Unilateral C/S |
| Danzig 2016 | Zno Group | Prob OUT only 2 - Danzig and Martinez | Post operative ECF Vs no ECF |
| Carter 2014 | Zno group | Prob OUT only 2 - Carter and Garvey | Mesh removal Vs no mesh removal |
| Hornby 2015 | Zno group | OUT - unclear whether absorbable/non-absorbable | Suture Vs no suture |
| Lorente-Herce 2015 | Zno group | OUT | Lower midline Vs Upper midline |
| Memon 2013 | Zno group | OUT | Midline Vs Pfannenstiel |
| Altom 2012 | ZNo group | OUT | Concomitant: same side Vs other side |
| Altom 2012 | ZNo group | OUT | Concomitant Vs Non-Concomitant |
| Azar 2017 | ZNo group | OUT | LOD >30% Vs LOD <30% |
| Basta 2015 | ZNo group | OUT | Estimated blood loss + ml Vs estimated blood loss |
| Bencini 2009 | ZNo group | OUT - only 1 | Hospital stay (Continuous variable, Multivariable HR analysis) |
| Bondre 2016 | ZNo group | OUT - only 2 | Skin flap Vs no Skin flap |
| Carter 2014 | ZNo group | OUT - only 1 | Institution 1 Vs Institution 2 |
| Carter 2014 | ZNo group | OUT - only 1 | Urine retention Vs no urine retention |
| Carter 2014 | ZNo group | OUT - only 1 | Ethnicity Afro-caribean Vs White caucassian |
| Carter 2014 | ZNo group | OUT - only 2 | Re-operation Vs no re-operation |
| Davidson 2009 | ZNo group | OUT - Probably not the same as pannicculectomy | Body contouring procedures Vs no body contouring procedures |
| Diamond 2015 | ZNo group | OUT - only 2 | Active mesh infection Vs no active infection (CDC4/VHWG4) |
| Dietz 2014 | ZNo group | OUT - only 1 | >=3 risk factors ref to 0-2 risk factors |
| Gecim 1996 | ZNo group | OUT - Constipation Vs no Constipation (ONLY 3) | Severe constipation Vs no severe constipation |
| Ghazi 2011 | ZNo group | OUT - only 1 | Risk factors >=2 Vs <2 |
| Giordano 2016 | ZNo group | OUT - only 1 | Mesh exposure Vs no mesh exposure |
| Giordano 2017c | ZNo group | OUT - only 1 | NSQUIP grade 3+4 Vs NSQUIP grade 1+2 |
| Greenstein 2008 | ZNo group | OUT - only 2 | Number of defects (</>=2) |
| Groene 2016b | ZNo group | OUT - only 1 | Symptomatic limited activity Vs non-symptomatic limited activity |
| Gronvold 2012 | ZNo group | OUT - only 1 | SingleSiteLap Vs StandardLap |
| Heimann 2017 | ZNo group | OUT - only 1 | No. of previous bowel resections+1 Vs no. of previous bowel resections |
| Helgstrand 2013 | ZNo group | OUT - only 1 | Longitudinal Vs Transverse incision |
| Hornby 2015 | ZNo group | OUT - only 1 | LOS >0 days Vs LOS 0 days |
| Hornby 2015 | ZNo group | OUT - only 1 | Use >3 ports Vs <=3 ports |
| Hultman 2014 | ZNo group | OUT - only 1 | Posterior release Vs no posterior release |
| Johnson 2016 | ZNo group | OUT - only 1 | Transabdominal Vs Total extraperitoneal approach |
| Kohler 2015a | ZNo group | OUT - only 1 | Coagulopathy Vs No coagulopathy |
| Kohler 2015a | ZNo group | OUT - only 1 | Rectus diastasis Vs no rectus diastasis |
| Korenkov 2002 | ZNo group | OUT - only 1 | Simple Hernia Vs Complex |
| Lahon 2009 | ZNo group | OUT - only 1 | Mesh size > 15x20 Vs Mesh size < 15x20 |
| Le Blanc 2003 | ZNo group | OUT - only 2 | Early Vs Late |
| Mercoli 2017 | ZNo group | OUT - only 1 | Clavien Dindo >2 Vs Clavien Dindo <=2 |
| Mercoli 2017 | ZNo group | OUT - only 1 | Clavien Dindo >2 Vs Clavien Dindo <=2 |
| Notash 2007 | ZNo group | OUT - only 1 | Mesh size+1cm^2 Vs mesh size |
| Renard 2017 | ZNo group | OUT - only 1 | Absorbable mesh Vs non absorbable mesh |
| Rinaldi 2016 | ZNo group | OUT - only 1 | Sarcopaenic Vs non-sarcopaenic patients |
| Slater 2015c | ZNo group | OUT - only 2 | No previous laparotomies+1 Vs No previous laparotomies |
| Slater 2015c | ZNo group | OUT - only 1 | Blood loss+500mls Vs blood loss |
| Slater 2015c | ZNo group | OUT - only 2 | Previous open abdomen Vs no previous open abdomen |
| Tollens 2011 | ZNo group | OUT - only 1 | Heavy manual work Vs no heavy manual work |
| Tollens 2011 | ZNo group | OUT - only 1 | Not another abdo wall hernia Vs presence of other abdo wall hernia |
| Tollens 2011 | ZNo group | OUT - only 1 | Not another abdo wall hernia Vs presence of other abdo wall hernia |
| Westen 2014 | ZNo group | OUT - only 1 | Chronic complaints Vs no chronic complaints |
| Wink 2014 | ZNo group | OUT - only 2 | Previous skin graft Vs previous primary closure |
| Wink 2014 | ZNo group | OUT - only 1 | Simultaneous stoma reversal Vs no simultaneous stoma reversal |
| Wink 2014 | ZNo group | OUT - only 1 | Previous mesh closure Vs previous primary closure |
| Wink 2014 | ZNo group | OUT - only 1 | Flap necrosis Vs no flap necrosis |
| Wink 2014 | ZNo group | OUT - only 2 | Traumatic hernia Vs non-traumatic hernia |
| Won 2015 | ZNo group | OUT - only 1 | Post operative hyperglycaemia Vs no post operative hyperglycaemia |
| Wormer 2016 | ZNo group | OUT - only 1 | indocyanine green vs no indocyanine green |

**Total 172 – deleted predictors**
