# Supplementary material for: Identifying predictors of ventral hernia recurrence: systematic review and meta-analysis
Source: BJS Open. 2021 Apr 11;5(2):zraa071. doi: 10.1093/bjsopen/zraa071 (PMC8038271; doi:10.1093/bjsopen/zraa071)
Supplement: zraa071_Supplementary_Data [file zraa071_supplementary_data.zip › OnlineResource9.Overall.docx]

**Online Resource 9**

| **Review question:** Overview of prognostic factors for recurrent ventral hernias  **Population:** Primary, Incisional or Primary and Incisional  **Prognostic factors:** Demographics, co-morbidities, hernia related, intra-operative and post-operative  **Studies:** RCTs, prospective cohorts, retrospective cohort, observational  **Meta-analysis estimates:** Univariate odds ratios were used | | | | | | | |
| --- | --- | --- | --- | --- | --- | --- | --- |
| **Prognostic factor** | **No. studies** | **No. patients** | **No. events** | | **Estimates reported** | **Meta-analysis estimate**  **(95%CI) [no. studies]** | **Notes** |
| ***Demographics*** | | | | | | | |
| Age | 25 | 34,484 | 3,995 | | 22 Uni OR  8 Multi OR  2 Uni HR  7 Multi HR  1 Multi RR | Age>60 Vs Age<60  0.86 (0.70 to 1.05) [7]  Age>65 Vs Age<65  0.72 (0.54 to 0.96) [7] | - Age thresholds in meta-analysis: results were presented from the most frequently used thresholds. Related thresholds were grouped - namely 55 and 60, 70 with 65. Other presentations with fewer results are not presented in meta-analysis i.e. continuous age and threshold of 50 years. - Some patients will be counted more than once in the total number of patients, as they are included in both the meta-analyses at the two age thresholds. - One article was excluded as patients overlapped with another article. |
| BMI | 49 | 14,454 | 1,703 | | 49 Uni OR  9 Multi OR  3 Uni HR  6 Multi HR  1 Multi RR | BMI>25 Vs BMI<25  2.51 (1.37 to 4.60) [5]  BMI>30 Vs BMI<30  1.54 (1.21 to 1.95) [25]  BMI>35 Vs BMI<35  1.42 (1.00 to 2.03) [9]  BMI>40 Vs BMI<40  3.07 (1.77 to 5.32) [4] | - BMI thresholds in meta-analysis: results were presented from the most frequently used thresholds. Related thresholds were grouped - namely 28 and 25, 32 with 30. Other presentations with fewer results are not presented in meta-analysis i.e. continuous BMI. - Some patients will be counted more than once in the total number of patients, as they are included in multiple meta-analyses at the different BMI thresholds. - Three articles were excluded as patients overlapped with other articles. |
| Sex | 43 | 14,948 | 1,987 | | 37 Uni OR  2 Multi OR  4 Uni HR  5 Multi HR | Male Vs Female  0.77 (0.61 to 0.97) [33] | - Three articles were excluded as patients overlapped with other articles. |
| ***Co-morbidities*** | | | | | | | |
| ASA | 16 | 5,828 | 1,002 | | 24 Uni OR  1 Multi OR  2 Uni HR  1 Multi HR | ASA 3-4 Vs ASA 1-2  1.46 (1.18 to 1.79) [11] | - ASA thresholds in meta-analysis: results were presented from the most frequently used threshold ASA 3-4 Vs ASA 2-1. Other presentations with fewer results are not presented in meta-analysis i.e. continuous ASA, ASA 2 Vs ASA 1 and ASA 4 Vs 3. For some articles different thresholds were combined in the comparison of ASA 3-4 Vs ASA 2-1. |
| BPH | 5 | 2,705 | 596 | | 5 Uni OR  1 Multi RR | BPH Vs No BPH  1.58 (0.74 to 3.36) [5] |  |
| Cardiac disease | 9 | 2,815 | 391 | | 8 Uni OR  2 Multi OR  1 Uni HR  1 Multi HR | Cardiac disease Vs No cardiac disease  1.49 (0.96 to 2.31) [8] |  |
| COPD | 22 | 5,910 | 932 | | 19 Uni OR  2 Multi OR  3 Uni HR  1 Multi HR | COPD Vs No COPD  1.53 (1.06 to 2.20) [16] | - Three articles were excluded as patients overlapped with other articles. |
| Diabetes | 28 | 6,064 | 1,095 | | 26 Uni OR  1 Multi OR  1 Uni HR | Diabetes Vs No Diabetes  1.36 (1.05 to 1.77) [25] | - One article was excluded as patients overlapped with another article. |
| Hypertension | 10 | 1,379 | 216 | | 10 Uni OR  1 Uni HR | Hypertension Vs No Hypertension  1.52 (0.88 to 2.62) [8] | - Two articles were excluded as patients overlapped with other articles. |
| Immunosuppression | 13 | 4,018 | 734 | | 13 Uni OR  1 Multi OR  1 Uni HR | Immunosuppression Vs No Immunosuppression  1.37 (0.72 to 2.59) [10]  Steroid use Vs No Steroid use  2.08 (1.08 to 3.98) [3] | - Some patients will be counted more than once in the total number of patients, as they are included in both the meta-analyses at the two thresholds. |
| Smoker | 32 | 14,454 | 1,307 | | 29 Uni OR  2 Multi OR  5 Uni HR  3 Multi HR | Smoker Vs Non-smoker  1.34 (1.03 to 1.74) [23] | - Smoker thresholds in meta-analysis: results were presented from the most frequently used threshold smoker vs non-smoker. Three studies using different thresholds were not included in the meta-analysis i.e. past smoker vs no smoker, active smoker vs non-active smoker and ex-smoker vs non-smoker. - Three articles were excluded as patients overlapped with other articles. |
| ***Hernia related*** | | | | | | | |
| AAA repair* | 3 | 721 | 152 | 3 Uni OR  1 Multi RR | | Previous AAA surgery Vs No Previous AAA surgery [3] | - No meta-analysis completed due to only three studies having AAA repair information - None of the studies indicated statistically significance difference between surgery methods. Two studies show increased odds of recurrence with AAA surgery compared to no AAA surgery; one study shows lower odds of recurrence. See Online resource 7 for individual study results. |
| Contaminated | 16 | 5,279 | 1,018 | 39 Uni OR  1 Multi OR  4 Uni HR | | Clean-contaminated Vs Clean0.67 (0.45 to 1.01) [7]  Contaminated Vs Clean 1.54 (0.68 to 3.49) [6]  Dirty Vs Clean  1.85 (0.77 to 4.43) [3]  Contaminated Vs Clean-contaminated  1.27 (0.61 to 2.67) [5]  Dirty Vs Clean-contaminated  1.85 (0.8 to 4.29) [4]  Dirty Vs Contaminated  0.69 (0.27 to 1.79) [4]  ECF repair Vs no ECF repair  3.09 (0.57 to 16.69) [3] | - Contamination thresholds in meta-analysis: results were presented from the most frequently used thresholds. Thresholds with fewer results are not presented in meta-analysis i.e. continuous estimates and all contaminated vs clean. - Some patients will be counted more than once in the total number of patients, as they are included in multiple meta-analyses at the different contamination thresholds. |
| Previous wound infection | 9 | 2,770 | 563 | 8 Uni OR  1 Multi OR | | Previous wound infection Vs no previous wound infection  1.43 (0.78 to 2.62) [8] |  |
| Hernia Area | 11 | 9,554 | 1,224 | 10 Uni OR  2 Multi OR  2 Uni HR  1 Multi HR  1 Multi RR | | Area>10cm^2^ Vs <10cm^2^  4.04 (0.77 to 21.27) [3]  Area >100cm^2^ Vs <100cm^2^  1.71 (0.76 to 3.85) [2] | - Hernia area thresholds in meta-analysis: results were presented from the most frequently used thresholds. Related thresholds were grouped – namely 25cm^2^ with 10cm^2^. Other presentations with fewer results are not presented in meta-analysis i.e. continuous area. For one article 20cm^2^ wasn’t used as the 10cm^2^ threshold was available. - Some patients will be counted more than once in the total number of patients, as they are included in both meta-analyses at the different thresholds. - One article was excluded as patients overlapped with another article. |
| Hernia Width | 36 | 49,471 | 5,001 | 46 Uni OR  14 MultiOR  3 Uni HR  8 Multi HR | | Width >2cm Vs <2cm  0.64 (0.24 to 1.72) [6]  Width >5cm Vs <5cm  1.09 (0.56 to 2.13) [11]  Width >10cm Vs <10cm  2.13 (0.89 to 5.09) [11]  Width >15cm Vs <15cm  2.33 (0.85 to 6.38) [7] | - Hernia width thresholds in meta-analysis: results were presented from the most frequently used thresholds. Related thresholds were grouped – namely hernia width 3cm was grouped with 2cm, defect width 4cm, 6cm, 7cm was grouped was with 5cm. Other presentations with fewer results are not presented in meta-analysis i.e. continuous hernia width and 20cm hernia width. - Some patients will be counted more than once in the total number of patients, as they are included in multiple meta-analyses at the different thresholds. - Three articles were excluded as patients overlapped with other articles. |
| Midline Vs Lateral | 14 | 3,391 | 427 | 11 Uni OR  2 Multi OR  1 Uni HR  2 Multi HR | | Midline Vs Lateral  1.00 (0.65 to 1.55) [10] | - One article was excluded as patients overlapped with another article. |
| Incisional Vs Primary | 23 | 12,299 | 1,543 | 22 Uni OR  5 Multi OR  1 Multi HR | | Incisional Vs Primary  1.79 (1.01 to 3.16) [18] | - Two articles were excluded as patients overlapped with other articles. |
| Recurrent Vs Primary | 52 | 56,548 | 11,223 | 55 Uni OR  11 MultiOR  6 Uni HR  3 Multi HR  1 Multi RR | | Recurrent Vs Primary  1.88 (1.48 to 2.40) [31] | - For the meta-analysis, results were presented from the most frequently used threshold recurrent vs primary. Other presentations with fewer results are not presented in meta-analysis i.e. continuous estimates and more than two hernia repairs vs less than two hernia repairs. - Six articles were excluded as patients overlapped with other articles. - One article had separate estimates for incisional only patients and primary and incisional patients which were kept separate for meta-analysis. - One article had different thresholds combined in the comparison of recurrent vs primary. |
| VHWG* | 7 | 5,020 | 739 | 28 Uni OR  2 Multi OR | | VHWG 3-4 Vs VHWG 1-2  2.04 (0.85 to 4.87) [4] | - No meta-analysis completed due to only four studies having VHWG information. - Two studies estimates showed statistically significance results, with increased odds of recurrence if have had VHWG 3-4 vs VHWG 1-2. One study shows higher odds of recurrence and another study shows lower odds of recurrence. See Online resource 7 for individual study results - One article was excluded as patients overlapped with another article. |
| ***Intra-operative*** | | | | | | | |
| Biological | 14 | 5,613 | 808 | | 16 Uni OR  2 Multi OR  5 Uni HR  2 Multi HR | Human Vs Porcine Mesh  1.59 (0.70 to 3.60) [5] | - For meta-analysis: Human vs xenograft, human vs bovine, porcine vs bovine subgroups were not included in meta-analysis due to overlapping data with other studies. - One article was excluded as patients overlapped with another article. |
| Biological Vs Synthetic | 19 | 3,786 | 742 | | 20 Uni OR  1 Multi OR  1 Multi HR | Biological Vs Synthetic Mesh  1.98 (1.22 to 3.22) [13]  Human Vs Synthetic Mesh0.9 (0.36 to 2.25) [3]  Porcine Vs Synthetic Mesh0.28 (0.07 to 1.12) [2] | - Some patients will be counted more than once in the total number of patients, as they are included in multiple meta-analyses at the different thresholds. - One article was excluded as patients overlapped with another article. |
| Bridging Vs Primary | 33 | 9,189 | 1,376 | | 30 Uni OR  3 Multi OR  2 Uni HR  4 Multi HR | Bridging Vs Primary fascial closure  2.62 (1.72 to 3.97) [27] | - Two articles were excluded as patients overlapped with other articles. |
| Component Separation | 18 | 3,944 | 461 | | 16 Uni OR  1 Multi OR  3 Uni HR  2 Multi HR | Component Vs No Component Separation  0.73 (0.49 to 1.10) [9]  Laparoscopic vs Open Component Separation  0.94 (0.54 to 1.64) [6] | - One article was excluded as patients overlapped with another article. |
| Concurrent GI* | 6 | 3,849 | 725 | | 5 Uni OR  2 Multi OR | Concurrent GI Vs No Concurrent GI [4] | - No meta-analysis completed due to only four studies having concurrent GI information. - One study estimate showed statistically significance results, with increased odds of recurrence if have had concurrent GI compared to no concurrent GI. - For the other studies, one showed increased odds and two showed lower odds of recurrence. See Online Resource 7 individual study results. - One article was excluded as patients overlapped with another article. |
| Lap Vs Open | 55 | 40,020 | 3,891 | | 55 Uni OR  2 Multi OR  1 Multi HR | Laparoscopic Vs Open surgery  0.76 (0.60 to 0.94) [48] | - Six articles were excluded as patients overlapped with other articles. - One article was excluded as there were no recurrences. |
| Mesh Weight | 7 | 2,084 | 142 | | 8 Uni OR | Lightweight Vs Heavyweight  1.46 (0.67 to 3.19) [4]  Lightweight Vs Mediumweight  1.62 (0.91 to 2.87) [3] | - Some patients will be counted more than once in the total number of patients, as they are included in both meta-analyses at the two thresholds. - One article was excluded as patients overlapped with another article. |
| Mesh Vs Suture | 55 | 43,587 | 5,515 | | 54 Uni OR  12 MultiOR  4 Multi HR  1 Multi RR | Mesh Vs Suture  0.66 (0.52 to 0.84) [48] | - For meta-analysis: biological and synthetic mesh was combined in the comparison of mesh vs suture. - Three articles were excluded as patients overlapped with other articles. |
| Mesh Position | 23 | 12,525 | 1,236 | | 42 Uni OR  3 Uni HR  1 Multi HR | Onlay Vs Retrorectus  1.96 (1.41 to 2.72) [9]  Onlay Vs Preperitoneal  1.46 (0.73 to 2.94) [4]  Onlay Vs Intraperitioneal  2.15 (0.87 to 5.34) [7]  Inlay Vs Onlay  1.25 (0.44 to 3.57) [5]  Inlay Vs Preperitoneal  1.6 (0.77 to 3.33) [3]  Inlay Vs Intraperitoneal  3.64 (0.87 to 15.14) [3]  Retrorectus Vs Intraperitoneal  0.42 (0.20 to 0.91) [6]  Preperitoneal Vs Intraperitoneal  2.94 (1 to 8.62) [3] | - Some patients will be counted more than once in the total number of patients, as they are included in multiple meta-analyses at the different thresholds. |
| Panniculectomy* | 6 | 1,409 | 151 | | 4 Uni OR  2 Uni HR | Panniculectomy Vs No Panniculectomy [4] | - No meta-analysis completed due to only four studies having panniculectomy information. - None of the studies indicated statistically significance difference between panniculectomy and no panniculectomy. Two studies show increased odds of recurrence with panniculectomy compared to no panniculectomy; one study shows no difference and one study showed lower odds of recurrence. See Online Resource 7 for individual study results |
| Tack Vs Suture* | 5 | 369 | 23 | | 5 Uni OR  1 Multi HR | Tack Vs Suture fixation [4] | - No meta-analysis completed due to only four studies having fixation information. - None of the studies indicated statistically significance difference between tack and suture fixation. Three studies show increased odds of recurrence with tack compared to suture and one study shows no difference in odds of recurrence. See Online Resource 7 for individual study results - One article was excluded as patients overlapped with another article. |
| ***Post-operative*** | | | | | | | |
| Complication | 8 | 4,433 | 445 | | 9 Uni OR  3 Multi OR  1 Uni HR  2 Multi HR | Complication Vs No Complication  3.34 (2.30 to 4.84) [6] | - For meta-analysis: small bowel obstruction, chest infection and urinary tract infection thresholds were not included due to few articles at these thresholds. |
| Seroma | 8 | 1,697 | 230 | | 8 Uni OR  1 Uni HR | Seroma Vs No Seroma  1.99 (1.22 to 3.24) [8] |  |
| SSO | 15 | 4,165 | 621 | | 12 Uni OR  1 Multi OR  5 Uni HR  1 Multi HR | SSO Vs No SSO  3.65 (2.40 to 5.56) [10]  Haematoma Vs No Haematoma  3.33 (1.33 to 8.33) [2] | - Some patients will be counted more than once in the total number of patients, as they are included in both meta-analyses at the two thresholds. |
| Wound Dehiscence | 5 | 2110 | 216 | | 4 Uni OR  1 Multi OR  1 Uni HR  1 Multi HR | Wound Dehiscence Vs No Wound Dehiscence  2.21 (1.2 to 4.06) [2] | - Two articles were excluded as patients overlapped with other articles. |
| Wound Infection | 31 | 8,563 | 1,500 | | 27 Uni OR  4 Multi OR  3 Uni HR  2 Multi HR  1 Multi RR | Wound Infection Vs No Wound Infection  3.21 (2.28 to 4.51) [24] | - For meta-analysis: wound infection with mesh infection and ECG threshold was not included due to few articles at this threshold. - Two articles were excluded as patients overlapped with other articles. |

* Prognostic factors were not included in meta-analysis, forest plots available in appendix
