# Supplementary material for: Identifying predictors of ventral hernia recurrence: systematic review and meta-analysis
Source: BJS Open. 2021 Apr 11;5(2):zraa071. doi: 10.1093/bjsopen/zraa071 (PMC8038271; doi:10.1093/bjsopen/zraa071)
Supplement: zraa071_Supplementary_Data [file zraa071_supplementary_data.zip › TitlePage.docx]

**Identifying Predictors of Ventral Hernia Recurrence: Systematic Review and Meta-Analysis**

Parker S.G^1^, Mallett S^2^, Quinn L^2^, Wood C.P.J^1^, Boulton R.W^1^, Jamshaid S^1^, Erotocritou M^3^, Gowda S^1^, Collier W^1^, Plumb A.A.O^4^, Windsor A.C.J^1^, Archer L^5^, Halligan S^4^.

Author affiliations:

1. The Abdominal Wall Unit, University College London Hospital, 235 Euston Road, London, NW1 2BU, UK.
2. Institute of Applied Health Research, Public Health Building, University of Birmingham, Birmingham, B15 2TT, UK.
3. UCL Medical School, 74 Huntley St, Bloomsbury, London WC1E 6DE, UK.
4. UCL Centre of Medical Imaging, 2^nd^ floor Charles Bell House, 43-45 Foley Street, W1W 7TS, UK.
5. Centre for Prognosis Research School of Primary, Community and Social Care, David Weatherall Building, Keele University, Staffordshire, ST5 5BG, UK.

**Corresponding author: Mr Samuel G Parker**

Address: The Abdominal Wall Unit, University College London Hospital, 235 Euston Road, London, NW1 2BU, UK.

Email: [samgparker@nhs.net](mailto:samgparker@nhs.net)

Mobile: 07814136705

**Author to whom requests for reprints should be sent: Mr Samuel G Parker**

Address: As above

**Funding**

This work was funded by the UK National Institute for Health Research (grant RfPB PB-PG-0816-20005) and Allergan PLC. Neither funders have been involved in the planning, methodology, analysis or write up of the research. National Institute of Health Research, Room 132, Richmond House, 79 Whitehall, London, SW1A 2NS. Allergan Plc, Clonshaugh Business and Technology Park, Coolock, Dublin, D17 E400, Ireland. Archer L was supported by an NIHR Research Methods Fellowship during her time at Birmingham University. Mallett S is supported by NIHR Birmingham Biomedical Research Centre at the University Hospitals Birmingham NHS Foundation Trust and the University of Birmingham. ﻿Halligan S is supported by the NIHR University College London Hospitals Biomedical Research Centre.

**Conflicts of interest**

A.C.J.W. declares conflicts of interest not directly related to the submitted work: consultant adviser for TELA BIO; educational grants and speaker for BARD, LifeCell and Cook. Parker S.G, Halligan S, Wood C.P.J, Boulton R.W, Jamshaid S, Erotocritou M, Gowda S, Collier W, Plumb A.A.O, Quinn L, Archer L, and Mallett S declare no conflict of interest.

**Manuscript category**

Review
